# A Single Base Change in the *csgD* Promoter Resulted in Enhanced Biofilm in Swine-Derived *Salmonella* Typhimurium

**DOI:** 10.3390/microorganisms12071258

**Published:** 2024-06-21

**Authors:** Zhe Li, Mengke Zhang, Gaopeng Lei, Xin Lu, Xiaorong Yang, Biao Kan

**Affiliations:** 1 National Key Laboratory of Intelligent Tracking and Forecasting for Infectious Diseases, National Institute for Communicable Disease Control and Prevention, Chinese Center for Disease Control and Prevention, Beijing 102206, China; lizhe@icdc.cn (Z.L.);; 2 School of Light Industry, Beijing Technology and Business University, Beijing 100048, China; 3 Center for Disease Control and Prevention of Sichuan Province, Chengdu 610041, China

**Keywords:** *Salmonella*, biofilm, *csgD*, host specificity, environmental adaptability

## Abstract

Pathogenic *Salmonella* strains causing gastroenteritis typically can colonize and proliferate in the intestines of multiple host species. They retain the ability to form red dry and rough (*rdar*) biofilms, as seen in *Salmonella enterica* serovar Typhimurium. Conversely, *Salmonella* serovar like Typhi, which can cause systemic infections and exhibit host restriction, are *rdar*-negative. In this study, duck-derived strains and swine-derived strains of *S*. Typhimurium locate on independent phylogenetic clades and display relative genomic specificity. The duck isolates appear more closely related to human blood isolates and invasive non-typhoidal *Salmonella* (iNTS), whereas the swine isolates were more distinct. Phenotypically, compared to duck isolates, swine isolates exhibited enhanced biofilm formation that was unaffected by the temperature. The transcriptomic analysis revealed the upregulation of *csgDEFG* transcription as the direct cause. This upregulation may be mainly attributed to the enhanced promoter activity caused by the G-to-T substitution at position −44 of the *csgD* promoter. Swine isolates have created biofilm polymorphisms by altering a conserved base present in *Salmonella* Typhi, iNTS, and most *Salmonella* Typhimurium (such as duck isolates). This provides a genomic characteristics perspective for understanding *Salmonella* transmission cycles and evolution.

## 1. Introduction

Salmonellosis caused by *Salmonella enterica* is a major foodborne disease that constitutes a significant global public health burden [1]. It poses a threat to the health of numerous hosts including animals, birds, fish, and humans [2]. The 2600 serotypes of the genus *Salmonella* exhibit considerable genetic diversity, enabling them to adapt to various environments and animal hosts [3]. The majority of human cases are caused by a few serotypes, which are loosely grouped into invasive/typhoidal (serovars Typhi and Paratyphi A) or non-typhoidal. Common non-typhoidal *Salmonella* (NTS) serotypes such as *S*. Typhimurium and *S*. Enteritidis typically cause self-limiting enterocolitis [4]. However, in regions with a high prevalence of immunosuppressive diseases, such as sub-Saharan Africa (sSA), NTS has become a primary cause of bacterial bloodstream infections [5] or invasive non-typhoidal *Salmonella* (iNTS) disease. A resistant biofilm physiology hypothesized that gastroenteritis-associated NTS retained the ability to form the red, dry, and rough (*rdar*) biofilm, while host-restricted *Salmonella* strains, such as *S*. Typhi and iNTS, lost the ability to form a *rdar* biofilm [3]. The evolution of *Salmonella* in balancing the tendency towards invasiveness and adaptability to different environmental niches remains an unresolved issue.

All *Salmonella* are transmitted through the fecal–oral route, and strains need to address issues related to interactions with hosts and the environment throughout their life cycle [6,7]. The formation of biofilms contributes to the environmental adaptability and persistence of *Salmonella* cells, especially NTS, during their transmission cycle [8]. As the preferred lifestyle for microorganisms in natural environments [9,10], biofilms constitute stable multicellular communities [11]. Cells embed themselves in the extracellular matrix they produce, facilitating their attachment to surfaces in their living environment. Compared to individual planktonic cells, biofilms exhibit higher resistance to host defenses [12], antimicrobial agents [13], desiccation [14], and disinfectants [15], and their physiological characteristics make them difficult to eradicate. The development of biofilm structures typically occurs in response to perceived negative environmental stimuli, such as changes in pH, temperature, oxygen, and nutrient availability [16,17]. In the complex structure of biofilms, microbial cells constitute only a small portion, with the majority of organic material forming the extracellular matrix, the composition of which can vary greatly depending on microbial and environmental factors [18]. Favorable conditions for biofilm formation include a low osmotic pressure medium and temperatures below 30 °C [19]. A nutrient limitation is known to activate polymer production, and various environmental signals can activate the transcription of *csgD*, which is part of the most complex regulatory network in *Salmonella* [18,20].

The colony biofilm formed by *S*. Typhimurium on Congo red (CR) agar are heterogeneous in structure and color [21,22]. The expression of the bacterial extracellular matrix components curli and cellulose leads to the *rdar* morphotype in biofilms [23]. The *rdar* biofilm on CR agar provides a good system for elucidating the molecular mechanisms of biofilm formation regulation, where changes in the expression of curli and cellulosecan can be visually observed through alterations in the morphology and color [24]. Various environmental factors (such as temperature, osmotic pressure, etc.) activate the expression of *csgD* through a complex regulatory network. CsgD is a member RpoS regulation and a major regulatory factor for the *rdar* morphotype [8,19]. Curli fimbriae [25], the biofilm-associated protein BapA [26], and cellulose [24] constitute the main structure of the *Salmonella* biofilm. *csgBAC*, *bapABCD*, *bcsABZC*, and *adrA* are key gene clusters and genes directing the synthesis of the above substances. *csgDEFG* are involved in the regulation and transport of these pathways. CsgD directly binds to the *csgBAC* promoter to activate curli synthesis and contributes indirectly to cellulose production by activating the transcription of *adrA* [27]. In addition, the synthesis of BapA [26] and the O-antigen capsule [28] also depends on CsgD (Figure S1). BapA is a ~386-kDa surface protein, comprising 27 tandem repeats predicted to be bacterial Ig-like (BIg) domains. These tandem repeat domains possess mechanical stability and are crucial for both early biofilm formation and mature biofilm integrity [29]. The genes for curli fimbriae and cellulose are highly conserved in *Salmonella* [30]. These two components act as the extracellular matrix scaffold, and their knockout will lead to single-cell behavior [31]. Currently, the relationship between the *Salmonella* biofilm morphology and pathogenic patterns has not been well explained at the genetic level. 

In this study, 18 strains of *S*. Typhimurium were isolated from farms in the Sichuan Province, China, with biological samples sourced from ducks and swine. According to their host sources, the isolated strains were located on two independent phylogenetic clades and displayed relative genomic specificity, potentially indicating host restriction. Duck isolates were more closely related evolutionarily to blood-derived strains, including iNTS. The biofilm morphology was a typical phenotypic difference between the two groups, with swine isolates having more wrinkled and stretchable biofilms, and being insensitive to temperature. We aimed to elucidate the guiding factors for biofilm transformation in *S*. Typhimurium at the genomic level. The G-to-T substitution at position −44 in the *csgD* promoter has been confirmed to enhance the biofilm morphology. This conservative base change increases the diversity of *Salmonella* biofilms in nature. This study will help deepen our understanding of the transmission cycle and evolution of *Salmonella* from a perspective of genomic characteristics.

## 2. Materials and Methods

### 2.1. Bacterial Strains, Plasmids, Primers, and Culture Conditions

The bacteria, plasmids, and primers used in this study are listed in Supplementary Tables S1–S4. Except for LT2, which is laboratory-preserved, all *Salmonella* Typhimurium strains involved in this study were isolated from biological samples, and their sources are provided in Table S1. *Escherichia coli* DH5α and SM10λpir were used for plasmid construction and suicide plasmid conjugation, respectively. The plasmids pWM91CM, pSRKGM, and pBBRlux were, respectively, used for homologous recombination, gene overexpression, and promoter activity evaluation. Unless otherwise specified, bacterial strains in this study were cultured in Lysogeny broth (LB) medium containing 1% NaCl at 37 °C. The final concentrations of antibiotics used during cultivation were as follows: Ampicillin (AMP, 100 μg mL^−1^), Gentamicin (GEN, 50 μg mL^−1^), chloramphenicol (CHL, 10 μg mL^−1^), Streptomycin (STR, 100 μg mL^−1^), and Kanamycin (KAN, 100 μg mL^−1^).

### 2.2. Construction of the Phylogenetic Tree

We collected a total of 471 genomes of *S*. Typhimurium from the EnteroBase database, with “Duck” (60), “Swine” (144), “Homo sapiens blood” (195), and “Homo sapiens feces” (72) as the keywords for “Source details” options. The “Uberstrain” numbers of the genomes were provided in Table S5. In addition, the genome of *S*. Typhimurium D23580 (GenBank accession GCA_000027025.1), a typical ST313 strain, was also included. We identified single-nucleotide polymorphisms (SNPs) between different bacterial strains using Snippy (v4.6.0). The reference genome was obtained from *S*. Typhimurium LT2, GenBank accession GCA_000006945.2, and SNPs within recombinant regions were excluded. To infer phylogenetic relationships, we used the maximum likelihood method of the GTR + I + G model with 1000 ultrafast bootstrap replications in IQ-TREE (v 2.0.4) [32]. Finally, we annotated and visualized the Newick files generated by IQ-TREE using iTOL (v 6.5.2) [33]. 

### 2.3. Induction of Biofilm Formation

Overnight cultured *S*. Typhimurium was adjusted to a McFarland density 4.0 (approximately OD_600_ 1.0) using fresh LB broth. In total, 5 μL of bacterial suspension was spotted onto the center of Congo Red LB agar without NaCl (40 μg mL^−1^ Congo Red, CAS: 573-58-0, Merck, Darmstadt, Germany) and incubated at 28 °C, 37 °C, and 42 °C for 72 h to observe colony morphology.

### 2.4. Crystal Violet Biofilm Assay

Overnight cultures of strains were diluted in fresh LB medium without NaCl and antibiotics to an OD_600_ 0.1. Ninety-six-well flat-bottom microtiter plates (Costar 3596; Cambridge, MA, USA) were inoculated with 200 μL of this suspension and incubated at 28 °C for 48 h with static culture. After incubation, liquid was removed from all wells and the wells were washed with sterile distilled water to remove any unbound cells. Biofilms were stained by adding 200 μL of 1% crystal violet (CAS: 548-62-9, Merck, Germany) to appropriate wells for 15 min. Crystal violet was removed, and each well was washed with sterile distilled water to remove unbound dye. The stained biofilm was dissolved by adding 200 μL of 70% ethanol, and the OD at 600 nm was measured using the Infinite 200 PRO (Tecan, Männedorf, Switzerland). All biofilm assays were performed with three biological replicates. Independent-sample *t*-tests were used to assess the significance of differences between samples.

### 2.5. Promoter Activity Assay

The upstream regulatory region sequences of *csgDEFG* and *csgBAC* from Sa5633 and Sa6866 were amplified by PCR and ligated between the *SacI* and *BamHI* restriction positions of the pBBRlux to generate recombinant luminescent reporter plasmids. *S*. Typhimurium LT2 was chosen as hosts for luminescent expression. A single colony was inoculated into LB medium (containing 10 μg mL^−1^ chloramphenicol and without NaCl) and cultured overnight at 37 °C until reaching the stationary phase. The culture was diluted to 1:100 in fresh LB medium as described above, and 200 μL of the bacterial suspension was inoculated into each well of a black 96-well plate (CAS: 3603, Corning, New York, NY, USA) and incubated statically at 28 °C. The luminescent signal was measured every 2 h using the Infinite 200 PRO (Tecan, Switzerland). Independent-sample *t*-tests were used to assess the significance of differences between samples.

### 2.6. Chromosomal Gene Editing

Gene editing was achieved using the suicide plasmid-mediated homologous recombination technique as described previously [34]. The suicide plasmid selected was the pWM91CM, derived from the pWM91 [35] and modified by inserting the promoter and ORF of the *cat* gene at the *BamHI* and *XhoI* positions. The 1 kb upstream and downstream regions of the target gene segment were amplified through PCR using genomes of Sa5633 or Sa6866 as a template, then combined by overlap PCR and inserted into the suicide plasmid. The cloned plasmid in *E. coli* SM10λpir was transformed into target strains. The transformants grown on salt-free sucrose plates which are chloramphenicol-sensitive are target clones. The deletion was confirmed through PCR screening and sequencing of the 2 kb amplicon produced by overlapping primers. PCR results were validated through sequencing. The primers used in this experiment are listed in Supplementary Tables S3.

### 2.7. Transcriptome Profiling

Scraped *S*. Typhimurium were cultured at 28 °C on biofilm-inducing plates (LB medium without NaCl) for 72 h, resuspended in PBS buffer, adjusted to an OD_600_ of 1.0, and then 1 mL of the bacterial suspension was quickly centrifuged to remove the supernatant. Total RNA was extracted using the Trizol method. In brief, 1 mL of Trizol solution (Invitrogen, CA, USA) was added to the bacterial cells, vigorously shaken for 15 s, and incubated at room temperature for 5 min. Then, 0.2 mL of chloroform was added to the mixture, vigorously shaken for 15 s, and then allowed to stand at room temperature for 3 min. After centrifugation at 12,000× *g* for 15 min at 4 °C, 400 μL of the supernatant was transferred to a new 1.5 mL Eppendorf (Ep) tube, followed by addition of an equal volume of isopropanol. After vigorous shaking to mix, the samples were left at room temperature for 10 min to allow RNA precipitation, followed by centrifugation at 12,000× *g* for 10 min at 4 °C to collect the RNA precipitate. Next, 1 mL of 75% ethanol was added to the precipitate, followed by centrifugation at 7500× *g* at 4 °C to remove the supernatant as much as possible. The RNA was air-dried at room temperature for 5 min until completely transparent and 50 μL of RNase-free water was added to dissolve the RNA in the precipitate, followed by the addition of DNase I (Invitrogen, Carlsbad, CA, USA) to remove genomic DNA. RNA quality was evaluated by agarose 1% gel electrophoresis, and the concentration was measured using a nanodrop spectrophotometer (Thermo Scientific, Waltham, MA, USA). RNA samples with A260/A280 > 1.9 and A260/A230 > 2 were selected for mRNA library preparation and Next-Generation Sequencing. Each sample was prepared with three biological replicates.

A total of 3 µg RNA per sample was utilized for RNA sample preparations. Sequencing libraries were generated with NEBNext^®^ Ultra™ Directional RNA Library Prep Kit (NEB, Ipswich, MA, USA) for Illumina^®^ (NEB, Ipswich, MA, USA) per manufacturer’s instructions. Index codes were added to associate sequences with each sample. mRNA was purified using poly-T oligo-attached magnetic beads (NEB, Ipswich, MA, USA), with rRNA removed. Fragmentation occurred using divalent cations at elevated temperature in NEBNext First-Strand Synthesis Reaction Buffer (5×) (NEB, Ipswich, MA, USA). First-strand cDNA was synthesized using random hexamer primer and M-MuLV Reverse Transcriptase (RNaseH-) (Invitrogen, Carlsbad, CA, USA). Second-strand cDNA synthesis used DNA Polymerase I (Invitrogen, Carlsbad, CA, USA) and RNase H, with dNTPs replaced by dUTP. Overhangs were converted into blunt ends via exonuclease/polymerase activities. Adenylation of 3’ ends of DNA fragments preceded ligation of NEBNext Adaptor with hairpin loop structure. cDNA fragments of 150~200 bp were selected and purified with AMPure XP system (Beckman Coulter, Beverly, WV, USA). USER Enzyme (NEB, USA) treated adaptor-ligated cDNA before PCR. PCR utilized Phusion High-Fidelity DNA polymerase, Universal PCR primers, and Index (X) Primer. Products were purified (AMPure XP system) and assessed on Agilent Bioanalyzer 2100 system. Clustering of index-coded samples was performed using TruSeq PE Cluster Kit v3-cBot-HS (Illumia, San Diego, CA, USA) on cBot Cluster Generation System. Library preparations were sequenced on Illumina Hiseq platform, generating paired end reads. Raw data (fastq format) underwent initial processing via in-house perl scripts. Clean data were obtained by removing adapter-containing reads, ploy-N reads, and low-quality reads. Q20, Q30, and GC content of clean data were calculated. Downstream analyses utilized clean data. Reference genome and gene model annotation files were obtained from genome website. Bowtie2-2.2.3 [36] built index of reference genome and aligned clean reads. DESeq R package (1.18.0) conducted differential expression analysis of two conditions/groups (two biological replicates per condition) using a negative binomial distribution model. *p*-values were adjusted using Benjamini and Hochberg’s approach. Genes with adjusted qvalue < 0.05 and a |log2(FoldChange)| > 1 from DESeq were considered differentially expressed.

### 2.8. Statistical Method

All statistical analyses in this study were performed using GraphPad Prism 9.0. For the two-sample unpaired *t*-test, two-tailed *p*-value and 95% confidence interval were selected. If the data variance is unequal, Welch’s correction can be applied. If the data do not follow a normal distribution, the Mann–Whitney test, a non-parametric test, can be used. *p*-values < 0.001 is defined as significant differences and is represented by “***”.

## 3. Results

### 3.1. SDSTs Exhibit Genomic Specificity towards DDSTs and an Enhanced Rdar Biofilm

**Figure 1:**
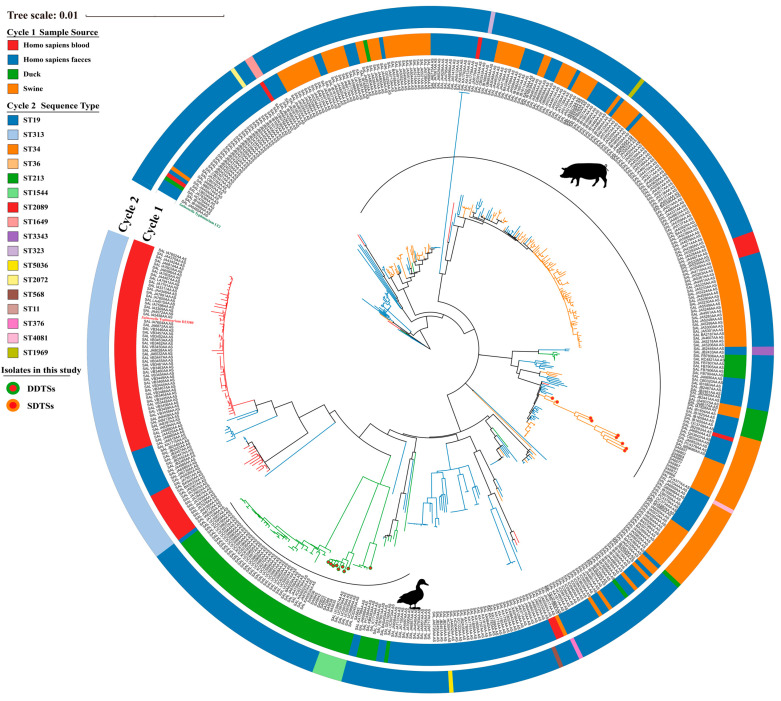
Phylogenetic relationships among *S*. Typhimurium strains isolated from ducks, swine, Homo sapiens blood, and Homo sapiens feces. *S*. Typhimurium D23580 is highlighted in red, while *S*. Typhimurium LT2 (reference) is highlighted in green.

In this study, nine strains each of duck-derived and swine-derived *S*. Typhimurium were isolated from farms in the Sichuan Province, China. They were designated as SDST (swine-derived *S*. Typhimurium) and DDST (duck-derived *S*. Typhimurium) based on their sources. We included genomes of *S*. Typhimurium (derived from ducks, swine, and Homo sapiens blood, and Homo sapiens feces) collected from the *EnteroBase* database to demonstrate their phylogenetic relationships with the isolated strains. Overall, DDSTs, SDSTs, and blood-derived strains each clustered together. DDSTs had a closer evolutionary relationship with iNTS (D23580) and the vast majority of blood-derived strains, indicating genomic similarity between them, while SDST is relatively independent (Figure 1). Fecal-derived strains exhibited a scattered distribution (Figure 1). Based on multilocus sequence typing (MLST), ST19 strains dominated numerically, covering almost all branches except for the blood-derived strains (Figure 1). Blood-derived strains, including the typical iNTS strain D23580, belonged to ST313 (Figure 1). The SDSTs isolated in this study all belonged to ST34 (Figure 1). Additionally, compared to genome clustering, the MLST was less precise in the classification. We observed that strains within the same cluster may belong to different ST groups (ST19 and ST1969), while the same ST strains may be located in different phylogenetic branches (ST19). Therefore, MLST cannot reflect the phylogenetic relationships among strains, making it difficult to establish accurate functional correlations between strains.

**Figure 2:**
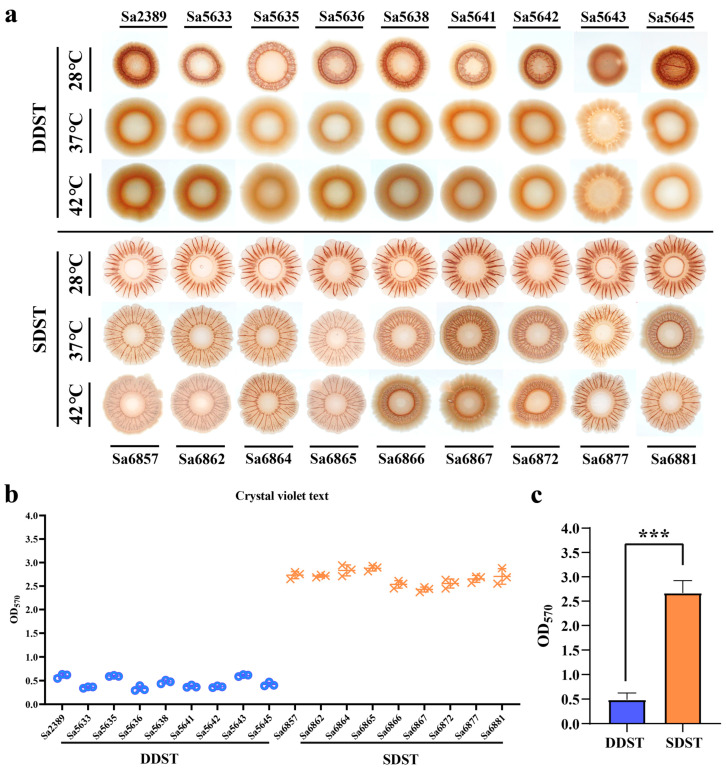
A comparison of biofilm formation between DDSTs and SDSTs. (**a**) Comparison of the biofilm morphology formed by DDSTs and SDSTs at three induction temperatures. Comparison of biofilm strength among individual isolates (**b**) and between the two groups (**c**) using crystal violet assay. Only the morphology of the biofilm is shown, and the size of the colonies is not displayed to scale. Three biological replicates were set up for each group, and “***” represents a *p*-value < 0.001.

We compared the biofilm morphology of DDSTs and SDSTs under a 28 °C induction temperature and their respective host body temperatures. DDSTs form red colonies with smooth centers and wrinkled edges at 28 °C (Figure 2a). In comparison, SDSTs formed colonies with more pronounced wrinkles and a larger extension of the edge (colony area not to scale), suggesting a stronger biofilm formation. Compared to *S*. Typhimurium ATCC 14028 [3,37], which was considered the standard for biofilms of this serotype, the biofilm of DDSTs were weakened, whereas that of SDSTs were enhanced. At 37 °C and 42 °C, DDST colonies became smooth and moist, with no noticeable wrinkles, indicating a significant reduction or loss of biofilm synthesis (Figure 2a). In the SDSTs, the wrinkling and extensibility of the biofilm decreased with the increasing temperature, but the *radr* phenotype was maintained (Figure 2a). Consequently, the biofilm of the SDST strains was superior to that of the DDST strains in terms of temperature stability and strength. Moreover, the biofilm advantages of the strains were not observed at the body temperatures of their respective hosts. We further quantitatively evaluated the strength of the biofilm at 28 °C through crystal violet text (Figure 2b). The results were consistent with the colony phenotype (Figure 2b). 

### 3.2. SNPs in bapA and bcsZ Cannot Cause Differences in Biofilm Morphology between DDSTs and SDSTs

We compared the sequence consistency of key genes involved in biofilm synthesis and regulatory networks between the two source strains, such as *csgBAC*, *csgDEFG*, *bcsABZC*, *adrA*, *rpoS*, *bapA*, *ompR,* etc. Multiple-sequence alignment revealed single-nucleotide polymorphisms (SNPs) located within the open reading frames (ORFs) of *bapA* and *bcsZ* between DDSTs and SDSTs. Three nucleotide changes were observed in *bapA*, including a G/A transition at position 1174, a C/A transversion at position 1795, and an A/G transition at position 3588 (Figure S2). The first two SNPs resulted in the conversion of glycine to serine and threonine to proline, respectively. The G to A transition at position 3588 replaced tryptophan with the stop codon TGA, leading to the premature termination of the *bapA* translation. BapA was divided into two parts, 1195 aa and 2558 aa. A C/T transition at position 277 was observed in the *bcsZ* (Figure S2), which is a member of the cellulose synthesis operon, leading to the substitution between tyrosine and histidine. 

**Figure 3:**
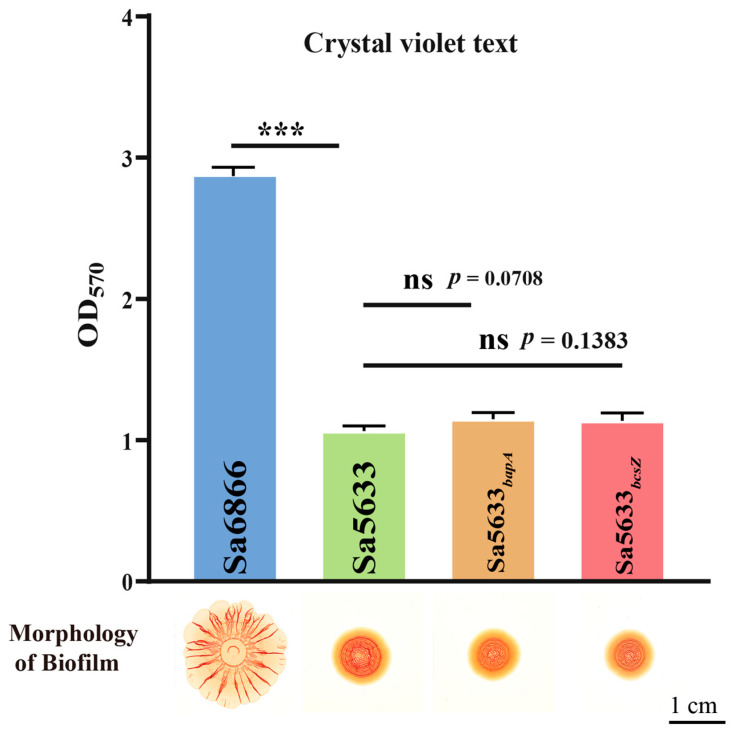
Evaluation of the impact of SNPs in *bapA* and *bcsZ* on biofilm formation. Three biological replicates were set up for each group. “ns” stands for “no significant difference” (*p*-value > 0.05) and “***” represents a *p*-value < 0.001.

Sa5633 and Sa6866 were selected as representative strains for SDSTs and DDSTs to evaluate the impact of these SNPs. The ORFs of *bapA* and *bcsZ* from Sa6866 were replaced by homologous recombination at the same locus in Sa5633, generating the recombinant strains Sa5633*_bapA_* and Sa5633*_bcsZ_*. Induced under 28 °C and no-NaCl cultivation conditions, Sa5633*_bapA_* and Sa5633*_bcsZ_* did not form the SDST-like *rdar* morphotype biofilm, and the crystal violet test showed no significant difference in biofilm formation and adhesion strength compared to Sa5633 (Figure 3). Therefore, these SNPs were not the cause of the biofilm differences.

### 3.3. RNA-seq Analysis Indicated That the Upregulation of csgDEFG and csgBAC May Be the Direct Cause of the Stronger Rdar Biofilm Formation in SDSTs

Excluding nucleotide changes in ORFs as a direct cause of biofilm differences, we explored genes with differential expression between DDSTs (Sa2389 and Sa5633) and SDSTs (Sa6866 and Sa6867) using RNA-seq. An average of 15 (14–16) million reads were generated for each biological replicate. Approximately, 95–98% of the reads mapped to the reference genome. The Pearson correlation coefficient (R^2^) between biological replicates ranged from 0.971 to 0.978.

**Figure 4:**
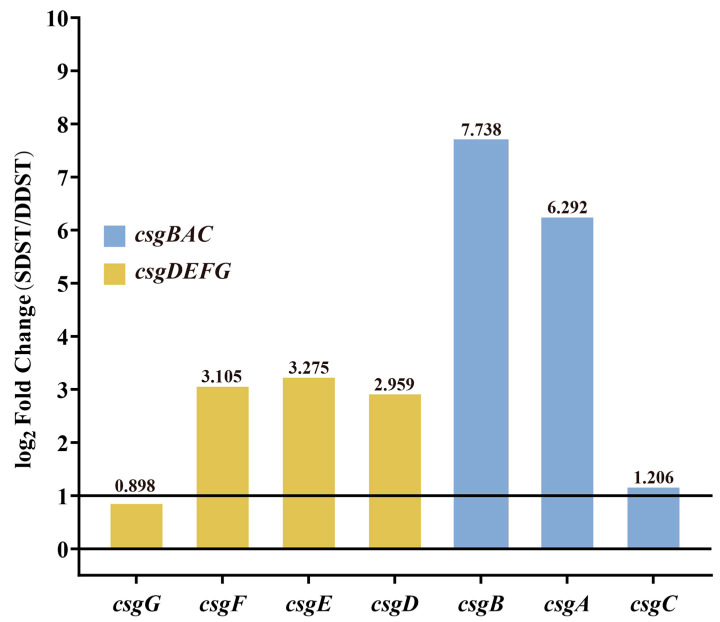
Differential expression of *csgDEFG* and *csgBAC* gene clusters between DDST and SDST groups. The cut-off for differential expression was set as qvalue < 0.05 and a |log_2_(FoldChange)| > 1. The values of log_2_Foldchange are indicated above the bars. DDST: duck-derived *S*. Typhimurium; SDST: swine-derived *S*. Typhimurium.

The cut-off for differential expression was set as q value < 0.05 and a |log_2_(FoldChange)| > 1. A total of 179 differentially expressed genes were identified in the transcriptome comparison between the SDST and DDST groups cultured under biofilm induction conditions, including 67 upregulated genes and 112 downregulated genes. Due to the more pronounced *rdar* biofilm morphology in SDSTs, attention was focused on genes that were upregulated in SDSTs. Five of the top ten genes (*csgB*, *csgA*, *csgE*, *csgF*, and *csgD*) with FoldChange were members of the *csgBAC* and *csgDEFG* operons (Figure 4, Table S6), suggesting that the upregulation of these two gene clusters may be the direct cause of the enhanced *radr* biofilm morphology. 

**Figure 5:**
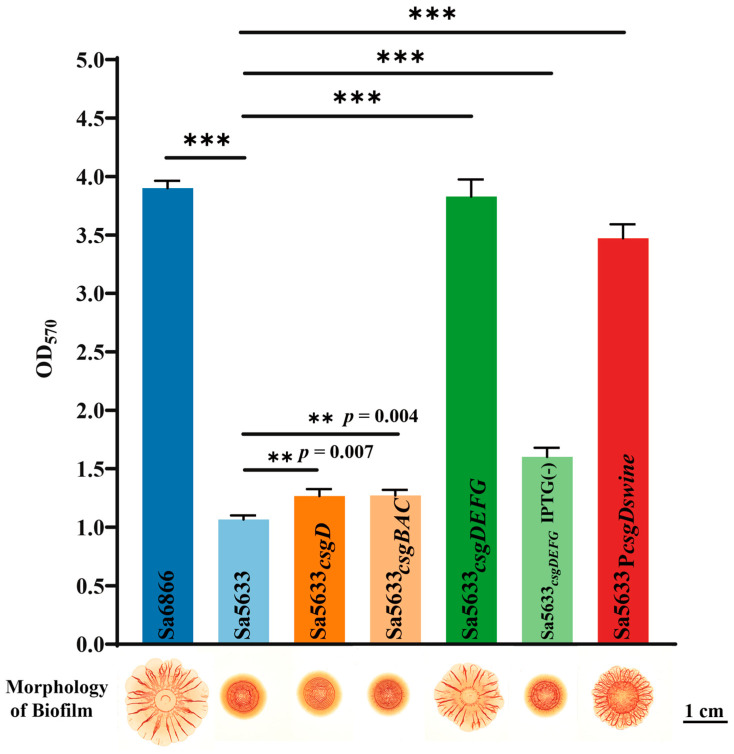
Changes in biofilm formation after overexpression of *csgD*, *csgDEFG*, *csgBAC*, and reversion of *csgD* promoter point mutations. Subscripts in the strain names indicate overexpression of this gene in the respective strain. Sa5633_P*csgDswine*_ was generated by replacing the upstream regulatory region (754 bp) of *csgD* in Sa5633 with the corresponding region from Sa6866. In crystal violet test, three biological replicates were set up for each group. “**” represents a *p*-value ≤ 0.01 and “***” represents a *p*-value < 0.001. Unless otherwise specified with IPTG (-), IPTG was added during cultivation by default.

To verify the relationship between the expression levels of *csgDEFG* and *csgBAC* and the biofilm formation ability, a recombinant plasmid based on pSRKGM was used to overexpress *csgD*, *csgDEFG*, and *csgBAC* in Sa5633, and the biofilm morphology of these overexpressed strains was observed. Only under IPTG induction, Sa5633*_csgDEFG_* formed the same *rdar* biofilm as Sa6866 (Figure 5). Merely increasing the expression level of CsgD was insufficient to alter the biofilm morphology (Figure 5). Although the overexpression of CsgD can enhance the transcription of *csgBAC*, it cannot alter the expression of its co-cluster genes *csgE*, *csgF*, and *csgG*. Therefore, the upregulation expression of *csgDEFG* was the direct cause of the enhanced *rdar* biofilm morphology, possibly resulting from mutations in its promoter region. The results of the crystal violet test were consistent with the biofilm morphology. The remaining genes with high differential expression have not been reported to be associated with biofilm formation in existing studies. 

### 3.4. The G to T Transversion at Position −44 in the csgD Promoter May Be the Main Cause for the Upregulation Expression of csgDEFG in SDSTs

**Figure 6:**
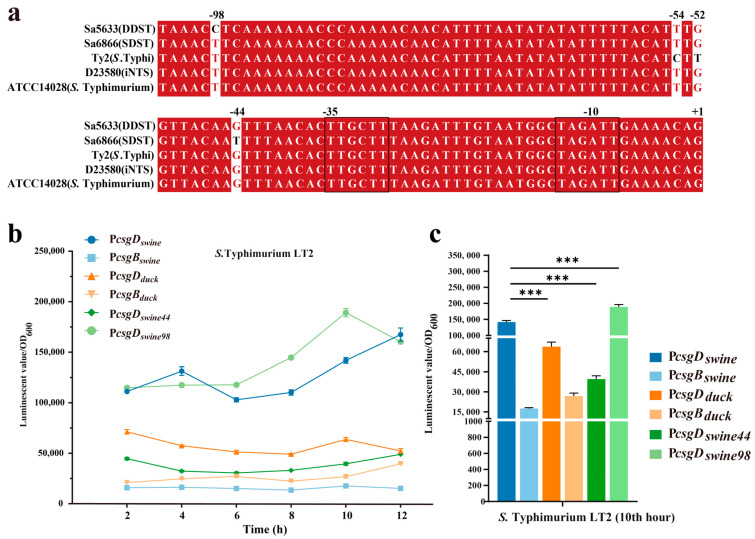
Multiple sequence alignment (**a**) and activity comparison (**b**,**c**) of the *csgD* promoter. Three biological replicates were set up for each group, and “***” represents a *p*-value < 0.001. The −10 and −35 regions of the promoter are outlined with black lines. Red bases and red fonts represent sequentially consistent sites, and black fonts represent different bases. (**b**) Time-course changes in promoter activity. (**c**) Comparison of promoter activity at the 10th hour. The sample names represent the types of promoters integrated before *luxCDABE* in pBBRlux plasmid; P*csgD_swine_*: *csgD* promoter from SDST; P*csgB_swine_*: *csgB* promoter from SDST; P*csgD_duc_*_k_: *csgD* promoter from DDST; P*csgB_duck_*: *csgB* promoter from DDST; P*csgD_swine44_*: T-to-G transversion at position −44 in P*csgD_swine_*; P*csgD_swine98_*: T-to-C transition at position −98 in P*csgD_swine_*.

Two base changes were observed in the intergenic regulatory region of the *csgD* and *csgB* genes at positions −44 (G/T) and −98 (C/T) of *csgD*, respectively (Figure 6a). To evaluate the impact of point mutations on promoter activity, the intergenic regions (754 bp) of *csgD* and *csgB* from Sa5633 and Sa6866 were cloned before the *luxCDABE* of the *pBBRlux* plasmid to evaluate promoter activity by the luminescent value (Figure 6b). Taking the light signal measured at 10 h as an example, the activity of the *csgD* promoter cloned from Sa6866 (P*csgD_swine_*) was significantly stronger than that cloned from Sa5633 (P*csgD_duck_*), while the activity of the *csgD* promoter (P*csgD_swine_* or P*csgD_duck_*) from the same source was stronger than that of the *csgB* promoter (P*csgB_swine_* or P*csgB_duck_*) (Figure 6c). This result is corroborated by the differential expression in the transcriptome (Figure 4 and Figure 6c). 

To assess the impact of each base mutation on the promoter activity, the single-mutation promoters P*csgD_swine44_* and P*csgD_swine98_* were constructed based on P*csgD_swine_*, corresponding to the T-to-G transversion at position −44 and the T-to-C transversion at position −98, respectively. P*csgD_swine44_* significantly reduced the activity of P*csgD_swine_*, while P*csgD_swine98_* exhibited increased transcriptional activity (Figure 6c). Therefore, it is speculated that the G-to-T transversion at position −44 of the *csgD* promoter may be the main cause for the upregulated expression of *csgDEFG* in SDSTs. By genetic recombination, the upstream regulatory region (754 bp) of the *csgD* in Sa6866 was replaced at the corresponding gene position in Sa5633, generating the Sa5633_P*csgDswine*_. At 28 °C, Sa5633_P*csgDswine*_ formed a *rdar* morphotype with significantly enhanced biofilm synthesis, although it did not fully reach the level of extension and wrinkling as Sa6866 (Figure 5). The Crystal Violet test was used to evaluate the adhesion of the biofilm, and the results were consistent with the colony morphology (Figure 5). Therefore, the transversion at position −44 in the promoter of the *csgD* directly affects the biofilm morphology. The G at position −44 was conserved among different biofilm phenotypes of *Salmonella*, such as *rdar*-deficient strains like *S*. Typhi and iNTS, and *rdar*-forming strains like DDSTs and *S*. Typhimurium ATCC14028 (Figure 6a). The G-to-T substitution at position −44 increased the strength of the *rdar* biofilm and also diversified the types of *Salmonella* biofilms in nature. Additionally, the T to C substitution at position −98 was unique to DDST, but it had little impact on the biofilm morphology.

## 4. Discussion

From a transmission perspective, the formation of biofilms is a conservative strategy that can increase the persistence and survival rate of pathogens. Up to 40% of zoonotic diseases are believed to be associated with biofilms, which have significant medical and economic implications [38,39]. In *Salmonella*, there is an association between biofilms and host intestinal colonization and adaptation. Biofilm formation is highly conserved in *Salmonella* strains associated with gastroenteritis, but it is lost in strains that cause invasive diseases or adapt to specific host lifestyles. Römling and his colleagues believe that the formation and loss of biofilms is a hallmark of pathogenic adaptation, potentially related to the ability to survive in host tissues [30]. A specific variant of the serovar Typhimurium strain (i.e., the Copenhagen variant) that causes systemic disease in pigeons has lost the ability to form the *rdar* morphotype [40]. A similar analysis was expanded to include other host-adapted serovars, such as Cholerasuis (swine) and Gallinarum (chickens), all of which were almost entirely *rdar* negative. 

One hypothesis is that the presence of an extracellular matrix seems to reduce the virulence of *Salmonella*, and the *rdar* biofilm may be a feature of weakened virulence. Curli represents a pathogen-associated molecular pattern (PAMP) that can activate Toll-like receptors 1 and 2 [41]. Cellulose has also been shown to play an important role in the interaction between the host and the pathogen during *Salmonella* infection. *Rdar*-negative strains may reduce the stimulation of the host immune system by losing such an extracellular matrix, making it easier to evade immune responses and thereby gaining advantages in adaptation and invasion within the host. Therefore, strains with weakened biofilms may receive more attention. In this study, we observed that swine-derived strains exhibit enhanced *rdar* biofilms, unlike typical *S*. Typhimurium. This enhancement manifests as a larger spreading area, more pronounced wrinkling, and a loss of temperature sensitivity. This provides the strain with greater stress resistance and an advantage in microenvironmental competition above 30 °C. On the other hand, this may also be an evolutionary direction more suited for colonization in the mammalian gut. Higher levels of an extracellular matrix may trigger a stronger immune response, leading to exacerbated gastroenteritis. It is noteworthy that bacterial biofilm communities are often composed of cells from multiple genera. The improvement in biofilm extensibility and strength can enhance the survival of resistant and pathogenic bacteria in the microenvironment, increasing pathogenic risk and treatment difficulty.

In most *Salmonella* and *E. coli* strains, *csgD* expression is activated at temperatures below 30 °C and inhibited at higher temperatures [19]. The *csgD* promoter region has single-nucleotide polymorphisms, resulting in dysregulated expression [42]. This study revealed that the thymine substitution at position −44 in the *csgD* promoter led to the upregulation of the *csgDEFG* and *csgBAC* transcription levels. When the −44 position of P*csgD_swine_* was replaced with guanine, the promoter activity significantly decreased. In *S*. Typhimurium var. Copenhagen isolates, the prevalent mutation was a G-to-T transversion in the −35 region in the *csgD* promoter, which may partially explain the loss of the biofilm phenotype [30]. CsgD is the primary transcriptional regulator of biofilm, controlling the synthesis of curli, cellulose, and other polymers in *Salmonella* and *E. coli* [43]. The expression of *csgD* is tightly repressed during the early stages of growth, but when cells enter the stationary phase, its expression is induced up to 370-fold [44]. In the stationary phase, cell density in the culture is high, nutrients become limited, and cell expression switches to regulation by the alternative sigma factor RpoS [45]. RpoS controls the general stress response [46] and selectively transcribes *csgD* [47]. Most of the regulatory proteins involved in *csgD* transcription regulation bind to two overlapping sites known as hotspot I and II in the intergenic region between *csgB* and *csgD* [48,49]. The −44 base of the *csgD* promoter is located in hotspot I, involving multiple regulatory protein-binding sites, such as the positive regulators OmpR, IHF, and the negative regulators CpxR, RstA, H-NS, etc., [50]. Competitive binding and cooperative binding exist between positive and negative regulatory factors [49,50], thus the upregulation of *csgD* transcription caused by the −44 position substitution may be the result of affecting the binding of one or multiple regulatory factors. Römling suggests that after this base substitution, *csgD* expression still requires OmpR, but the sigma factor required for *csgD* promoter expression changes from σ^S^ to the housekeeping σ factor σ^D^ [19]. This view is now widely accepted. Generally, *csgD* regulates various phenotypes, including biofilm, in a dynamic bistable expression pattern [51]. This can be seen as a bacterial bet-hedging strategy against unknown environmental stresses, ensuring that at least one group of cells is better suited to the encountered survival conditions by controlling *csgD* to be ON or OFF. Therefore, when the promoter shifts to a like-constitutive type that is insensitive to environmental inducing factors, some stress resistance will be lost. The constant expression of biofilm will inevitably consume bacterial nutrients, depleting the expression of pathogenic-related functions. In a mouse co-infection model, planktonic cells consistently maintained dominance [52].

Phylogenetically, DDSTs show higher genomic similarity to blood-derived strains. Compared to *S*. Typhimurium ATCC 14028, DDSTs exhibited a weakened *rdar* morphology. This leads us to speculate that DDSTs are also similar to iNTS in terms of biological functions, such as invasiveness. iNTS strains, DDSTs, and SDSTs present three models of *S*. Typhimurium biofilms, ranging from loss to continuous synthesis. We also observed a significant motility advantage of SDSTs at 37 °C and 42 °C (data not provided), which may contribute to its biofilm extensibility. We endowed DDST with the *csgD* promoter from SDST, and although the wrinkling and extensibility were enhanced, they did not reach the levels of SDST. Considering the genomic specificity of the two source strains, there are still factors affecting the biofilm morphology that need to be further explored.

## 5. Conclusions

This study reports that DDSTs and SDSTs isolated from farms in the Sichuan Province, China, have genomic specificity, and DDST is more closely related to blood-derived strains (such as iNTS). Phenotypically, the biofilm of SDSTs exhibits significantly greater wrinkles and expansiveness compared to DDSTs, and is not dependent on temperature. We confirmed that the upregulation of *csgDEFG* expression is the direct cause of the difference, and the G-to-T transversion at position −44 of the *csgD* promoter is its genetic basis. After the base substitution, the biofilm of DDST was reinforced. The biofilm morphology guided by this base transversion enriches the biofilm diversity of *Salmonella* under natural conditions, which will help understand the survival adaptation and evolution of *Salmonella* from the perspective of genomic characteristics.

## Data Availability

All data needed to evaluate the conclusions in this paper are present in the paper and/or the Supplementary Materials. Additional data related to this paper may be requested from the authors. Correspondence and requests for materials should be addressed to B.K. (kanbiao@icdc.cn).

## Supplementary Materials

The following supporting information can be downloaded at: https://www.mdpi.com/article/10.3390/microorganisms12071258/s1, Figure S1: The biofilm formation and regulatory network mediated by *csgD* in *Salmonella*; Figure S2: Multiple sequence alignment of the *bapA* (a) and *bcsZ* (b) in DDSTs and SDSTs; Table S1: Sample information of 18 *S*. Typhimurium isolates; Table S2: Engineering bacteria, standard strains, and genetically modified strains in this study; Table S3: Plasmids used in this study; Table S4: Primers used in this study; Table S5: The genomes of *S*. Typhimurium used in the phylogenetic analysis; Table S6: Genes upregulated in the SDST group compared to the DDST group in the transcriptome analysis. References [53,54,55] are cited in the Supplementary Materials.
