# Supplementary material for: A Single Base Change in the csgD Promoter Resulted in Enhanced Biofilm in Swine-Derived Salmonella Typhimurium
Source: Microorganisms. 2024 Jun 21;12(7):1258. doi: 10.3390/microorganisms12071258 (PMC11278976; doi:10.3390/microorganisms12071258)
Supplement: Supplementary file 1 [file microorganisms-12-01258-s001.zip › microorganisms-3001620-supplementary.pdf]

Supplementary Data for

A Single Base Change in the *csgD* Promoter Resulted in Biofilm Defects in Duck-Derived *Salmonella* Typhimurium

Zhe Li *et al.*

*Corresponding author. B.K. (Email: kanbiao@icdc.cn)

This PDF file includes:

Figs. S1 to S2
Tables S1 to S6
References (1-4)

SUPPLEMENTARY DATA

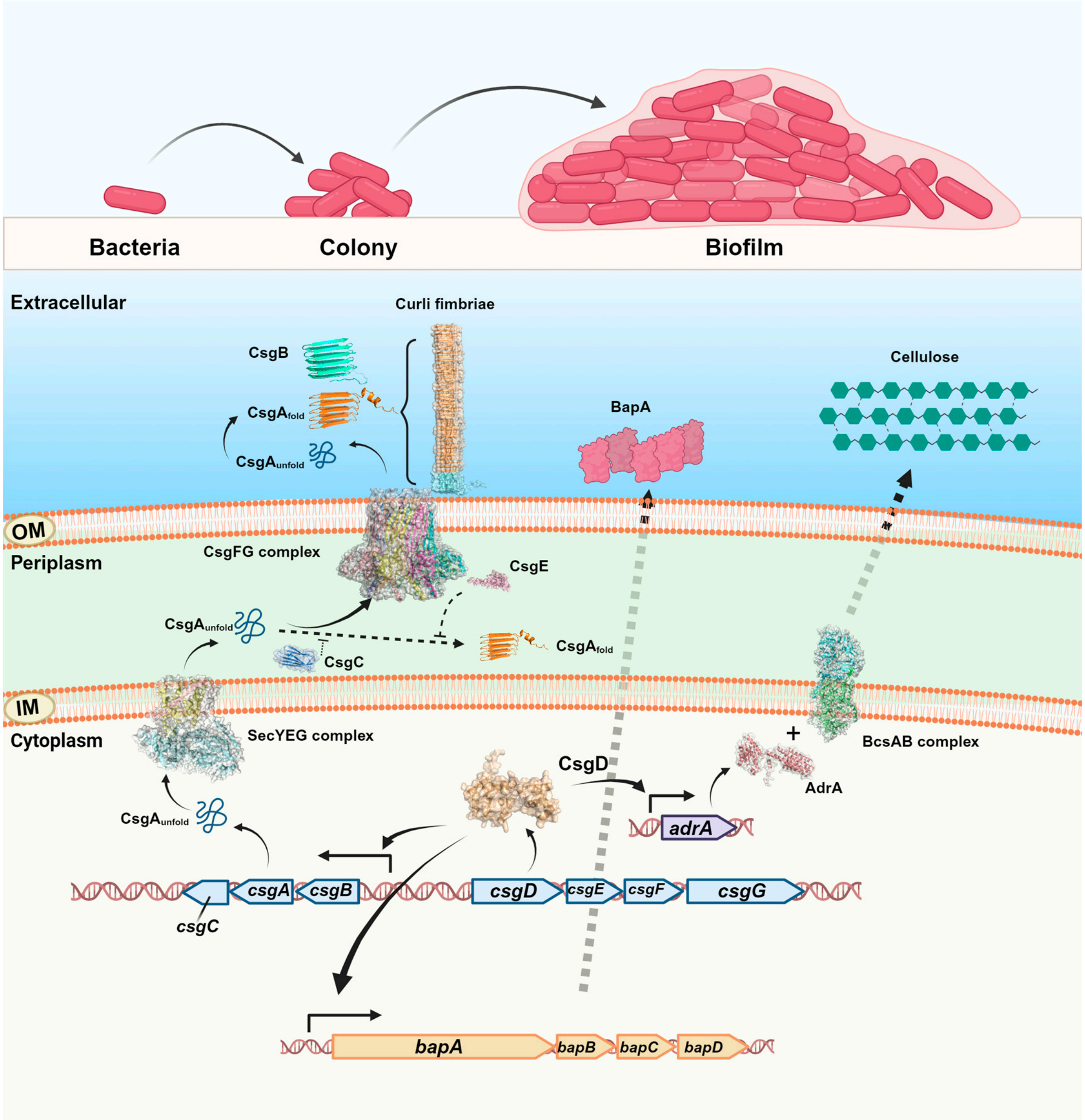

Figure S1 The biofilm formation and regulatory network mediated by *csgD* in *Salmonella*. The schematic diagram was created with BioRender.com.

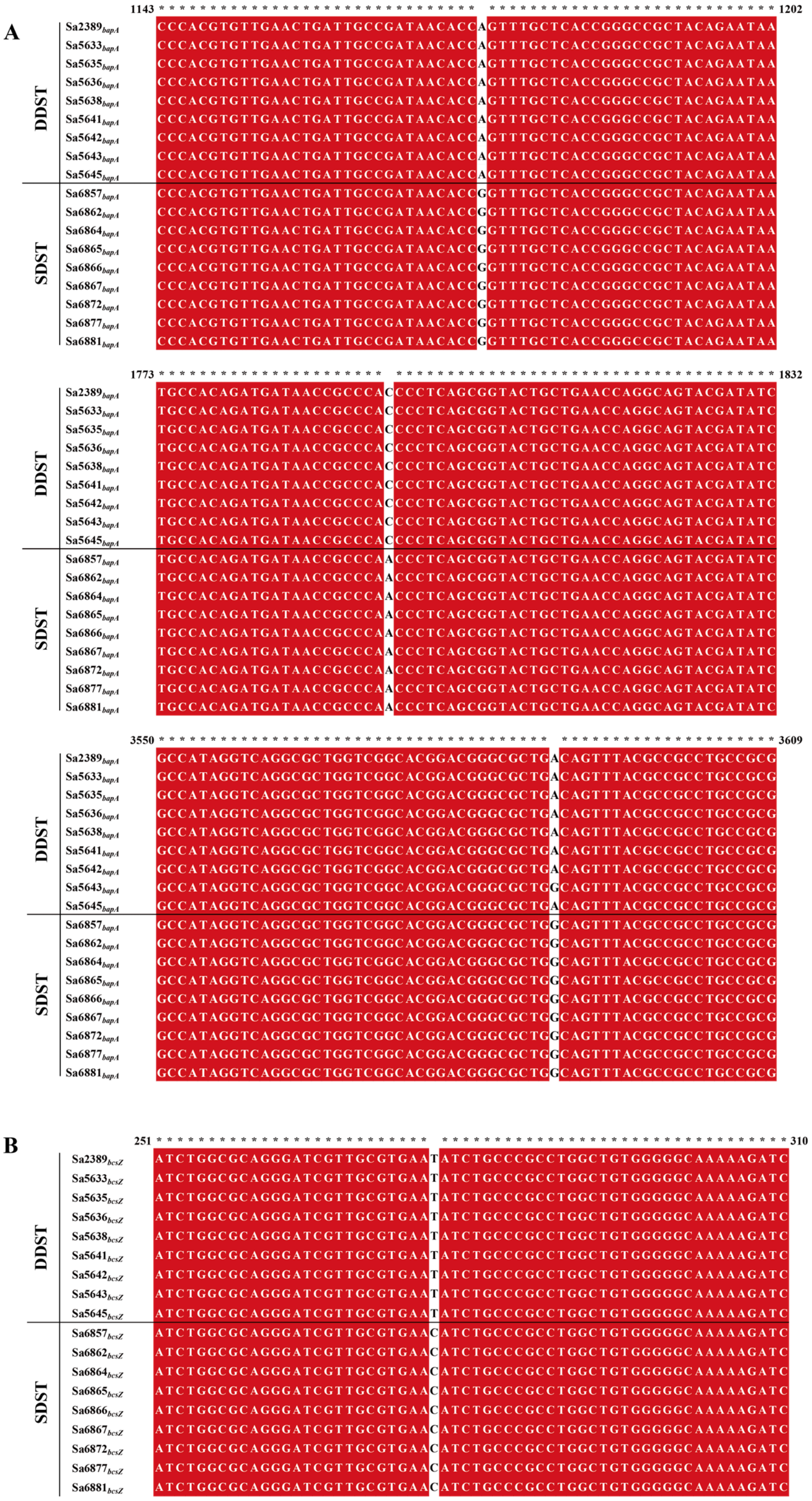

Figure S2 Multiple sequence alignment of the *bapA* (a) and *bcsZ* (b) in DDSTs and SDSTs. Numbers represent the start and end positions of sequence alignment."*" is marked on completely identical nucleotide positions.

Table S1

Sample information of 18 *S.* Typhimurium isolates

Strains	Host	Sample type	Sampling times
Sa2389	Duck	Preserved egg	2008
Sa5633	Duck	Duck (carcass)	2011
Sa5635	Duck	Duck egg	2011
Sa5636	Duck	Duck egg	2011
Sa5638	Duck	Duck egg	2012
Sa5641	Duck	Duck (Anal swab)	2011
Sa5642	Duck	Duck (Anal swab)	2012
Sa5643	Duck	Duck (Anal swab)	2011
Sa5645	Duck	Duck (Anal swab)	2011
Sa6857	Swine	Pig (ileocecal forest knot)	2011
Sa6862	Swine	Pig (Surface swab of carcass)	2011
Sa6864	Swine	Pig (Surface swab of carcass)	2011
Sa6865	Swine	Pig (Anal swabs)	2011
Sa6866	Swine	Pig (ileocecal forest knot)	2011
Sa6867	Swine	Pig (Anal swabs)	2011
Sa6872	Swine	Pig (Anal swabs)	2011
Sa6877	Swine	Pig (Anal swabs)	2011
Sa6881	Swine	Pig (Surface swab of carcass)	2011

Table S2

Engineering bacteria, standard strains, and genetically modified strains in this study

Strains	Genotype or phenotype	References
E. coli SM10 λ*pir*	*thi thr leu tonA lacY supE recA::RP4-2-Tc::Mu Km* λ*pir*	(1)
E. coli DH5α	F- φ80 lacZΔM15 Δ(lacZYA-argF)U169 *recA1 endA1 hsd*R17(rk^-, mk^+) *phoA*, supE44 thi-1 *gyrA96 relA1* λ^-	Lab collections stock
S. Typhimurium LT2	ST19; STR^R	Lab collections stock
$Sa5633_{PcsgDswine}$	Sa5633 recombined the upstream regulatory region (754 bp) of *csgD* from Sa6866	Lab collections stock
$Sa5633_{bapA}$	Sa5633 recombined *bapA* from Sa6866	Lab collections stock
$Sa5633_{bcsA}$	Sa5633 recombined *bcsA* from Sa6866	Lab collections stock
$Sa5633_{csgD}$	Sa5633 carrying pSRKGM-CsgD	Lab collections stock
$Sa5633_{csgBAC}$	Sa5633 carrying pSRKGM-CsgBAC	Lab collections stock
$Sa5633_{csgDEFG}$	Sa5633 carrying pSRKGM-CsgDEFG	Lab collections stock

Table S3

Plasmids used in this study

Plasmids	Genotype or phenotype	References
pWM91	Suicide plasmid; *oriV*$_{R6K\gamma}$ *oriT lacZα traJ sacB*; Amp^R	(2)
pWM91CM	Suicide plasmid; *oriV*$_{R6K\gamma}$ *oriT lacZα traJ sacB*; Amp^R Cm^R	This study
pWM91CM-*bapA*	pWM91CM carrying *bapA* of Sa6866	This study
pWM91CM-*bcsZ*	pWM91CM carrying *bcsZ* of Sa6866	This study
pWM91CM-*PcsgD*	pWM91 carrying the *csgD* and *csgB* intergenic region (754bp) and upstream and downstream 1000 bp gene fragments	This study
pBBRlux	bioluminescence based reporter plasmid containing a promoterless *luxCDABE* operon; CmR	(3)
P*csgD*$_{swine}$	pBBRlux inserted upstream regulatory region of *csgD* from Sa6866 before *luxCDABE* operon	This study
P*csgB*$_{swine}$	pBBRlux inserted upstream regulatory region of *csgB* from Sa6866 before *luxCDABE* operon	This study
P*csgD*$_{duck}$	pBBRlux inserted upstream regulatory region of *csgD* from Sa5633 before *luxCDABE* operon	This study
P*csgB*$_{duck}$	pBBRlux inserted upstream regulatory region of *csgB* from Sa5633 before *luxCDABE* operon	This study
P*csgD*$_{swine}$ -44 T>G	-44 T to G transversion in P*csgD*$_{swine}$	This study
P*csgD*$_{swine}$ -98 T>C	-98 T to C transition in P*csgD*$_{swine}$	This study
pSRKGM	expression vector containing the P_{lac} promoter; $LacI^q$ repressor, *lacZα*$^+$:MCS, pBBR1 ori; Gm^r	(4)
pSRKGM-CsgD	pSRKGM with insertion of *csgD* gene	This study
pSRKGM-CsgBAC	pSRKGM with insertion of *csgBAC* gene cluster	This study
pSRKGM-CsgDEFG	pSRKGM with insertion of *csgDEFG* gene cluster	This study

Table S4

Primers used in this study

**Primers**	**Sequence (5'–3')**
REC*bapA*-up-F	CAGTGAAGTGCTTCATGTGGATCCATGCGTCTACTCGCCGTGGTTTCGAAATTG
REC*bapA*-down-F	CTAAAGGGAACAAAAGCTGGAGCTCTCAACCGCTGGTTATCAGGTGG
REC*bcsZ*-up-F	CAGTGAAGTGCTTCATGTGGATCCCACTTTATCGCGATGCCGGATTTAC
REC*bcsZ*-down-F	CTAAAGGGAACAAAAGCTGGAGCTCCGATAGAGCGACTGACGGACTAAG
RECP*csgD*-up-F	CAGTGAAGTGCTTCATGTGGATCCGCGGCAAGGAGCAATAAAGTATGC
RECP*csgD*-down-F	CTAAAGGGAACAAAAGCTGGAGCTCGCGTTTGATCTATTTGTCGGCG
Lux-PcsgD-F	CTCACTATAGGGCGAATTGGAGCTCAGCGAAAATTATCTATTACCTTGTTAG
Lux-PcsgD-R	GCGGCCGCAACTAGAGGATCCGATGAAACTCCACTTTTTTAATCGCAC
Lux-PcsgB-F	CTCACTATAGGGCGAATTGGAGCTCGATGAAACTCCACTTTTTTAATCGCAC
Lux-PcsgB-R	GCGGCCGCAACTAGAGGATCCAGCGAAAATTATCTATTACCTTGTTAG
GM-CsgD-F	CACTATAGGGCGAATTGGGTACCATGTTTAATGAAGTCCATAGTAGTCATG
GM-CsgD-R	GCAGGAATTCGATATCAAGCTTTTACCGCCTGAGATTATCGTTTG
GM-CsgBAC-F	CACTATAGGGCGAATTGGGTACCATGTACGACCAGGTCCAGGGTGAC
GM-CsgBAC-R	GCAGGAATTCGATATCAAGCTTCTACTGTGCAGAAGGCGGCCATTG
GM-CsgDEFG-F	CACTATAGGGCGAATTGGGTACCATGTTTAATGAAGTCCATAGTAGTCATG
GM-CsgDEFG-R	GCAGGAATTCGATATCAAGCTTTCAGGATTCTGGCGGTACTGACAGC
M13F	CAGGAAACAGCTATGACC
M13R	TGTAAAACGACGGCCAGT

Table S5

The genomes of *Salmonella* Typhimurium used in the phylogenetic analysis. (1)

Genome sequence name	Uberstrain	Sample source	Genome sequence name	Uberstrain	Sample source
SAL_CA4304AA_AS	SAL_BA1416AA	Duck	SAL_IB1689AA_AS	SAL_UA7512AA	Homo sapiens feces
SAL_BA6209AA_AS	SAL_DA0311AA	Duck	SAL_IB1686AA_AS	SAL_UA7515AA	Homo sapiens feces
SAL_JA4728AA_AS	SAL_DA0336AA	Duck	SAL_IB1685AA_AS	SAL_UA7516AA	Homo sapiens feces
SAL_JA6766AA_AS	SAL_DA0337AA	Duck	SAL_IB1684AA_AS	SAL_UA7517AA	Homo sapiens feces
SAL_JA6385AA_AS	SAL_DA2007AA	Duck	SAL_IB1683AA_AS	SAL_UA7518AA	Homo sapiens feces
SAL_JA8797AA_AS	SAL_EA5272AA	Duck	SAL_IB1682AA_AS	SAL_UA7519AA	Homo sapiens feces
SAL_KA0052AA_AS	SAL_EA6534AA	Duck	SAL_IB1678AA_AS	SAL_UA7523AA	Homo sapiens feces
SAL_KA0053AA_AS	SAL_EA6535AA	Duck	SAL_IB1677AA_AS	SAL_UA7524AA	Homo sapiens feces
SAL_FB7906AA_AS	SAL_TA7563AA	Duck	SAL_IB1676AA_AS	SAL_UA7525AA	Homo sapiens feces
SAL_FB7905AA_AS	SAL_TA7564AA	Duck	SAL_IB1668AA_AS	SAL_UA7533AA	Homo sapiens feces
SAL_FB7904AA_AS	SAL_TA7565AA	Duck	SAL_IB1667AA_AS	SAL_UA7534AA	Homo sapiens feces
SAL_FB7903AA_AS	SAL_TA7566AA	Duck	SAL_IB1665AA_AS	SAL_UA7536AA	Homo sapiens feces
SAL_FB7902AA_AS	SAL_TA7567AA	Duck	SAL_IB1664AA_AS	SAL_UA7537AA	Homo sapiens feces
SAL_FB7901AA_AS	SAL_TA7568AA	Duck	SAL_IB1663AA_AS	SAL_UA7538AA	Homo sapiens feces
SAL_FB7807AA_AS	SAL_TA7647AA	Duck	SAL_IB1660AA_AS	SAL_UA7541AA	Homo sapiens feces
SAL_FB7806AA_AS	SAL_TA7648AA	Duck	SAL_IB1659AA_AS	SAL_UA7542AA	Homo sapiens feces
SAL_IB8152AA_AS	SAL_VA1501AA	Duck	SAL_IB1657AA_AS	SAL_UA7544AA	Homo sapiens feces
SAL_IC1716AA_AS	SAL_WA2839AA	Duck	SAL_IB1656AA_AS	SAL_UA7545AA	Homo sapiens feces
SAL_KB8383AA_AS	SAL_WA2874AA	Duck	SAL_IB1655AA_AS	SAL_UA7546AA	Homo sapiens feces
SAL_HC4764AA_AS	SAL_CB7540AA	Duck	SAL_IB1654AA_AS	SAL_UA7547AA	Homo sapiens feces
SAL_MC8566AA_AS	SAL_FB8402AA	Duck	SAL_IB1653AA_AS	SAL_UA7548AA	Homo sapiens feces
SAL_PC4384AA_AS	SAL_HB0720AA	Duck	SAL_IB1652AA_AS	SAL_UA7549AA	Homo sapiens feces
SAL_RC4821AA_AS	SAL_JB3012AA	Duck	SAL_IB1650AA_AS	SAL_UA7551AA	Homo sapiens feces
SAL_UC5953AA_AS	SAL_MB1957AA	Duck	SAL_IB1648AA_AS	SAL_UA7553AA	Homo sapiens feces
SAL_UC5954AA_AS	SAL_MB1958AA	Duck	SAL_IB1627AA_AS	SAL_UA7574AA	Homo sapiens feces
SAL_UC5955AA_AS	SAL_MB1959AA	Duck	SAL_IB1625AA_AS	SAL_UA7576AA	Homo sapiens feces
SAL_UC5956AA_AS	SAL_MB1960AA	Duck	SAL_IB1621AA_AS	SAL_UA7580AA	Homo sapiens feces
SAL_UC5957AA_AS	SAL_MB1961AA	Duck	SAL_IB1619AA_AS	SAL_UA7582AA	Homo sapiens feces
SAL_UC5958AA_AS	SAL_MB1962AA	Duck	SAL_IB1617AA_AS	SAL_UA7584AA	Homo sapiens feces
SAL_UC5959AA_AS	SAL_MB1963AA	Duck	SAL_IB1615AA_AS	SAL_UA7586AA	Homo sapiens feces
SAL_UC5960AA_AS	SAL_MB1964AA	Duck	SAL_IB1614AA_AS	SAL_UA7587AA	Homo sapiens feces
SAL_UC5961AA_AS	SAL_MB1965AA	Duck	SAL_IB1611AA_AS	SAL_UA7590AA	Homo sapiens feces
SAL_UC5962AA_AS	SAL_MB1966AA	Duck	SAL_IB1609AA_AS	SAL_UA7592AA	Homo sapiens feces
SAL_UC5963AA_AS	SAL_MB1967AA	Duck	SAL_IB1606AA_AS	SAL_UA7595AA	Homo sapiens feces
SAL_UC5964AA_AS	SAL_MB1968AA	Duck	SAL_IB1597AA_AS	SAL_UA7604AA	Homo sapiens feces
SAL_UC5965AA_AS	SAL_MB1969AA	Duck	SAL_JB2467AA_AS	SAL_VA6373AA	Homo sapiens feces
SAL_UC5966AA_AS	SAL_MB1970AA	Duck	SAL_JB2465AA_AS	SAL_VA6375AA	Homo sapiens feces

The genomes of *Salmonella* Typhimurium used in the phylogenetic analysis. (2)

Genome sequence name	Uberstrain	Sample source	Genome sequence name	Uberstrain	Sample source
SAL_UC5967AA_AS	SAL_MB1971AA	Duck	SAL_JB2461AA_AS	SAL_VA6379AA	Homo sapiens feces
SAL_UC5968AA_AS	SAL_MB1972AA	Duck	SAL_JB2460AA_AS	SAL_VA6380AA	Homo sapiens feces
SAL_UC5969AA_AS	SAL_MB1973AA	Duck	SAL_JB2457AA_AS	SAL_VA6383AA	Homo sapiens feces
SAL_UC5971AA_AS	SAL_MB1975AA	Duck	SAL_JB2456AA_AS	SAL_VA6384AA	Homo sapiens feces
SAL_UC5972AA_AS	SAL_MB1976AA	Duck	SAL_JB2453AA_AS	SAL_VA6387AA	Homo sapiens feces
SAL_UC5973AA_AS	SAL_MB1977AA	Duck	SAL_JB2452AA_AS	SAL_VA6388AA	Homo sapiens feces
SAL_UC5976AA_AS	SAL_MB1980AA	Duck	SAL_JB2451AA_AS	SAL_VA6389AA	Homo sapiens feces
SAL_UC5977AA_AS	SAL_MB1981AA	Duck	SAL_JB2450AA_AS	SAL_VA6390AA	Homo sapiens feces
SAL_UC5978AA_AS	SAL_MB1982AA	Duck	SAL_JB2449AA_AS	SAL_VA6391AA	Homo sapiens feces
SAL_UC5979AA_AS	SAL_MB1983AA	Duck	SAL_JB2448AA_AS	SAL_VA6392AA	Homo sapiens feces
SAL_UC5980AA_AS	SAL_MB1984AA	Duck	SAL_JB2447AA_AS	SAL_VA6393AA	Homo sapiens feces
SAL_UC5981AA_AS	SAL_MB1985AA	Duck	SAL_JB2446AA_AS	SAL_VA6394AA	Homo sapiens feces
SAL_UC5982AA_AS	SAL_MB1986AA	Duck	SAL_JB2445AA_AS	SAL_VA6395AA	Homo sapiens feces
SAL_UC5983AA_AS	SAL_MB1987AA	Duck	SAL_JB2444AA_AS	SAL_VA6396AA	Homo sapiens feces
SAL_UC5984AA_AS	SAL_MB1988AA	Duck	SAL_JB2443AA_AS	SAL_VA6397AA	Homo sapiens feces
SAL_XC6183AA_AS	SAL_PB0254AA	Duck	SAL_JB2441AA_AS	SAL_VA6399AA	Homo sapiens feces
SAL_XC6186AA_AS	SAL_PB0257AA	Duck	SAL_JB2440AA_AS	SAL_VA6400AA	Homo sapiens feces
SAL_XC6190AA_AS	SAL_PB0261AA	Duck	SAL_JB2439AA_AS	SAL_VA6401AA	Homo sapiens feces
SAL_XC6203AA_AS	SAL_PB0274AA	Duck	SAL_JB2438AA_AS	SAL_VA6402AA	Homo sapiens feces
SAL_XC6236AA_AS	SAL_PB0307AA	Duck	SAL_JB2437AA_AS	SAL_VA6403AA	Homo sapiens feces
SAL_XC6245AA_AS	SAL_PB0316AA	Duck	SAL_JB2436AA_AS	SAL_VA6404AA	Homo sapiens feces
SAL_XC6270AA_AS	SAL_PB0341AA	Duck	SAL_JB2435AA_AS	SAL_VA6405AA	Homo sapiens feces
SAL_XC6279AA_AS	SAL_PB0350AA	Duck	SAL_JB2434AA_AS	SAL_VA6406AA	Homo sapiens feces
SAL_IA5458AA_AS	SAL_AA8723AA	Homo sapiens blood	SAL_JB2433AA_AS	SAL_VA6407AA	Homo sapiens feces
SAL_IA3309AA_AS	SAL_BA7854AA	Homo sapiens blood	SAL_JB2432AA_AS	SAL_VA6408AA	Homo sapiens feces
SAL_IA3311AA_AS	SAL_BA7857AA	Homo sapiens blood	SAL_JB2431AA_AS	SAL_VA6409AA	Homo sapiens feces
SAL_IA7597AA_AS	SAL_BA7862AA	Homo sapiens blood	SAL_JB2430AA_AS	SAL_VA6410AA	Homo sapiens feces
SAL_IA7598AA_AS	SAL_BA7864AA	Homo sapiens blood	SAL_JB2429AA_AS	SAL_VA6411AA	Homo sapiens feces
SAL_IA7600AA_AS	SAL_BA7866AA	Homo sapiens blood	SAL_JB2428AA_AS	SAL_VA6412AA	Homo sapiens feces
SAL_IA7602AA_AS	SAL_BA7868AA	Homo sapiens blood	SAL_JB2427AA_AS	SAL_VA6413AA	Homo sapiens feces
SAL_IA7603AA_AS	SAL_BA7870AA	Homo sapiens blood	SAL_JB2426AA_AS	SAL_VA6414AA	Homo sapiens feces
SAL_IA7604AA_AS	SAL_BA7872AA	Homo sapiens blood	SAL_JB2425AA_AS	SAL_VA6415AA	Homo sapiens feces
SAL_IA7605AA_AS	SAL_BA7874AA	Homo sapiens blood	SAL_JB2424AA_AS	SAL_VA6416AA	Homo sapiens feces
SAL_IA8393AA_AS	SAL_CA0588AA	Homo sapiens blood	SAL_JB2423AA_AS	SAL_VA6417AA	Homo sapiens feces
SAL_IA8394AA_AS	SAL_CA0590AA	Homo sapiens blood	SAL_JB2422AA_AS	SAL_VA6418AA	Homo sapiens feces
SAL_IA8397AA_AS	SAL_CA0594AA	Homo sapiens blood	SAL_JB2421AA_AS	SAL_VA6419AA	Homo sapiens feces
SAL_JA6880AA_AS	SAL_CA0596AA	Homo sapiens blood	SAL_JB2420AA_AS	SAL_VA6420AA	Homo sapiens feces
SAL_IA8409AA_AS	SAL_CA0606AA	Homo sapiens blood	SAL_HC5047AA_AS	SAL_DB1433AA	Homo sapiens feces

The genomes of *Salmonella* Typhimurium used in the phylogenetic analysis. (3)

Genome sequence name	Uberstrain	Sample source	Genome sequence name	Uberstrain	Sample source
SAL_IA8413AA_AS	SAL_CA0613AA	Homo sapiens blood	SAL_HC5048AA_AS	SAL_DB1434AA	Homo sapiens feces
SAL_JA8159AA_AS	SAL_DA4752AA	Homo sapiens blood	SAL_PC4385AA_AS	SAL_HB0715AA	Homo sapiens feces
SAL_JA8028AA_AS	SAL_DA4758AA	Homo sapiens blood	SAL_OC3749AA_AS	SAL_HB0716AA	Homo sapiens feces
SAL_JA8180AA_AS	SAL_DA4781AA	Homo sapiens blood	SAL_OC3748AA_AS	SAL_HB0717AA	Homo sapiens feces
SAL_JA7851AA_AS	SAL_DA4838AA	Homo sapiens blood	SAL_PC4380AA_AS	SAL_HB0727AA	Homo sapiens feces
SAL_JA6532AA_AS	SAL_DA4879AA	Homo sapiens blood	SAL_PC4379AA_AS	SAL_HB0728AA	Homo sapiens feces
SAL_BA5533AA_AS	SAL_DA4903AA	Homo sapiens blood	SAL_PC4378AA_AS	SAL_HB0729AA	Homo sapiens feces
SAL_BA5445AA_AS	SAL_DA4932AA	Homo sapiens blood	SAL_PC4377AA_AS	SAL_HB0730AA	Homo sapiens feces
SAL_JA4342AA_AS	SAL_DA7437AA	Homo sapiens blood	SAL_OC3734AA_AS	SAL_HB0731AA	Homo sapiens feces
SAL_JA4572AA_AS	SAL_DA7438AA	Homo sapiens blood	SAL_UC7223AA_AS	SAL_MB2988AA	Homo sapiens feces
SAL_JA4407AA_AS	SAL_DA7439AA	Homo sapiens blood	SAL_CD1940AA_AS	SAL_RB9644AA	Homo sapiens feces
SAL_JA6872AA_AS	SAL_DA7440AA	Homo sapiens blood	SAL_BD0231AA_AS	SAL_RB9680AA	Homo sapiens feces
SAL_JA4370AA_AS	SAL_DA7442AA	Homo sapiens blood	SAL_BD0232AA_AS	SAL_RB9681AA	Homo sapiens feces
SAL_LA4015AA_AS	SAL_DA7443AA	Homo sapiens blood	SAL_GD3192AA_AS	SAL_WB2820AA	Homo sapiens feces
SAL_JA4346AA_AS	SAL_DA7444AA	Homo sapiens blood	SAL_GD3292AA_AS	SAL_WB2870AA	Homo sapiens feces
SAL_JA6596AA_AS	SAL_DA7445AA	Homo sapiens blood	SAL_GD3914AA_AS	SAL_WB3863AA	Homo sapiens feces
SAL_JA6869AA_AS	SAL_DA7594AA	Homo sapiens blood	SAL_BA2187AA_AS	SAL_BA3466AA	Swine
SAL_JA7838AA_AS	SAL_DA7597AA	Homo sapiens blood	SAL_BA3346AA_AS	SAL_BA3467AA	Swine
SAL_JA4517AA_AS	SAL_DA7599AA	Homo sapiens blood	SAL_BA3995AA_AS	SAL_BA3484AA	Swine
SAL_JA6367AA_AS	SAL_DA7600AA	Homo sapiens blood	SAL_BA4181AA_AS	SAL_BA3485AA	Swine
SAL_JA6550AA_AS	SAL_DA7602AA	Homo sapiens blood	SAL_BA5329AA_AS	SAL_BA3506AA	Swine
SAL_JA6772AA_AS	SAL_DA7603AA	Homo sapiens blood	SAL_BA5395AA_AS	SAL_BA3529AA	Swine
SAL_JA7962AA_AS	SAL_DA7605AA	Homo sapiens blood	SAL_BA5628AA_AS	SAL_BA3534AA	Swine
SAL_JA7934AA_AS	SAL_DA7606AA	Homo sapiens blood	SAL_BA5799AA_AS	SAL_BA3543AA	Swine
SAL_JA7891AA_AS	SAL_DA7609AA	Homo sapiens blood	SAL_BA5804AA_AS	SAL_BA3544AA	Swine
SAL_JA6814AA_AS	SAL_DA7610AA	Homo sapiens blood	SAL_BA6112AA_AS	SAL_BA3559AA	Swine
SAL_JA7847AA_AS	SAL_DA7611AA	Homo sapiens blood	SAL_BA6133AA_AS	SAL_BA3622AA	Swine
SAL_JA7791AA_AS	SAL_DA7612AA	Homo sapiens blood	SAL_BA6168AA_AS	SAL_BA3633AA	Swine
SAL_JA4425AA_AS	SAL_DA7614AA	Homo sapiens blood	SAL_CA4004AA_AS	SAL_BA3649AA	Swine
SAL_JA7978AA_AS	SAL_DA7615AA	Homo sapiens blood	SAL_HC8630AA_AS	SAL_BA4859AA	Swine
SAL_JA8176AA_AS	SAL_DA7616AA	Homo sapiens blood	SAL_IA5972AA_AS	SAL_BA5444AA	Swine
SAL_LA7941AA_AS	SAL_GA1353AA	Homo sapiens blood	SAL_IA5973AA_AS	SAL_BA6152AA	Swine
SAL_MA6426AA_AS	SAL_GA8166AA	Homo sapiens blood	SAL_IA5977AA_AS	SAL_BA8814AA	Swine
SAL_JB2093AA_AS	SAL_VA6641AA	Homo sapiens blood	SAL_IA5982AA_AS	SAL_CA0396AA	Swine
SAL_VB3468AA_AS	SAL_BB0916AA	Homo sapiens blood	SAL_IA5983AA_AS	SAL_CA7088AA	Swine
SAL_VB3467AA_AS	SAL_BB0917AA	Homo sapiens blood	SAL_IA5984AA_AS	SAL_CA7584AA	Swine
SAL_VB3466AA_AS	SAL_BB0918AA	Homo sapiens blood	SAL_IA5986AA_AS	SAL_CA7648AA	Swine
SAL_VB3464AA_AS	SAL_BB0920AA	Homo sapiens blood	SAL_IA6000AA_AS	SAL_CA7777AA	Swine

The genomes of *Salmonella* Typhimurium used in the phylogenetic analysis. (4)

Genome sequence name	Uberstrain	Sample source	Genome sequence name	Uberstrain	Sample source
SAL_VB3463AA_AS	SAL_BB0921AA	Homo sapiens blood	SAL_IA6006AA_AS	SAL_CA7780AA	Swine
SAL_VB3462AA_AS	SAL_BB0922AA	Homo sapiens blood	SAL_IA6017AA_AS	SAL_CA7781AA	Swine
SAL_VB3461AA_AS	SAL_BB0923AA	Homo sapiens blood	SAL_IA6173AA_AS	SAL_CA7786AA	Swine
SAL_VB3460AA_AS	SAL_BB0924AA	Homo sapiens blood	SAL_IA6364AA_AS	SAL_CA7787AA	Swine
SAL_VB3459AA_AS	SAL_BB0925AA	Homo sapiens blood	SAL_IA6483AA_AS	SAL_CA7788AA	Swine
SAL_VB3458AA_AS	SAL_BB0926AA	Homo sapiens blood	SAL_IA7828AA_AS	SAL_CA7789AA	Swine
SAL_VB3457AA_AS	SAL_BB0927AA	Homo sapiens blood	SAL_IA8354AA_AS	SAL_CA7790AA	Swine
SAL_VB3456AA_AS	SAL_BB0928AA	Homo sapiens blood	SAL_JA0295AA_AS	SAL_CA7980AA	Swine
SAL_VB3455AA_AS	SAL_BB0929AA	Homo sapiens blood	SAL_JA4331AA_AS	SAL_CA7984AA	Swine
SAL_VB3454AA_AS	SAL_BB0930AA	Homo sapiens blood	SAL_JA4357AA_AS	SAL_CA8278AA	Swine
SAL_VB3453AA_AS	SAL_BB0931AA	Homo sapiens blood	SAL_JA4359AA_AS	SAL_CA8336AA	Swine
SAL_VB3452AA_AS	SAL_BB0932AA	Homo sapiens blood	SAL_JA4361AA_AS	SAL_CA8533AA	Swine
SAL_VB3450AA_AS	SAL_BB0934AA	Homo sapiens blood	SAL_JA4390AA_AS	SAL_CA8777AA	Swine
SAL_VB3449AA_AS	SAL_BB0935AA	Homo sapiens blood	SAL_JA4420AA_AS	SAL_CA8782AA	Swine
SAL_VB3448AA_AS	SAL_BB0936AA	Homo sapiens blood	SAL_JA4431AA_AS	SAL_CA8798AA	Swine
SAL_VB3447AA_AS	SAL_BB0937AA	Homo sapiens blood	SAL_JA4451AA_AS	SAL_CA8818AA	Swine
SAL_VB3446AA_AS	SAL_BB0938AA	Homo sapiens blood	SAL_JA4460AA_AS	SAL_CA8836AA	Swine
SAL_VB3445AA_AS	SAL_BB0939AA	Homo sapiens blood	SAL_JA4463AA_AS	SAL_CA8991AA	Swine
SAL_VB3444AA_AS	SAL_BB0940AA	Homo sapiens blood	SAL_JA4467AA_AS	SAL_CA9122AA	Swine
SAL_IA6371AA_AS	SAL_BA5505AA	Homo sapiens feces	SAL_JA4471AA_AS	SAL_CA9310AA	Swine
SAL_JA6529AA_AS	SAL_DA3036AA	Homo sapiens feces	SAL_JA4475AA_AS	SAL_CA9322AA	Swine
SAL_JA4487AA_AS	SAL_DA3149AA	Homo sapiens feces	SAL_JA4481AA_AS	SAL_CA9323AA	Swine
SAL_JA5147AA_AS	SAL_DA3159AA	Homo sapiens feces	SAL_JA4488AA_AS	SAL_CA9518AA	Swine
SAL_JA7118AA_AS	SAL_DA3177AA	Homo sapiens feces	SAL_JA4496AA_AS	SAL_CA9520AA	Swine
SAL_JA0412AA_AS	SAL_DA3179AA	Homo sapiens feces	SAL_JA4500AA_AS	SAL_CA9529AA	Swine
SAL_JA4994AA_AS	SAL_DA3192AA	Homo sapiens feces	SAL_JA4514AA_AS	SAL_DA0195AA	Swine
SAL_JA7061AA_AS	SAL_DA3193AA	Homo sapiens feces	SAL_JA4528AA_AS	SAL_DA2321AA	Swine
SAL_JA6957AA_AS	SAL_DA3299AA	Homo sapiens feces	SAL_JA4571AA_AS	SAL_DA2325AA	Swine
SAL_JA6584AA_AS	SAL_DA3330AA	Homo sapiens feces	SAL_JA4589AA_AS	SAL_DA2352AA	Swine
SAL_JA4793AA_AS	SAL_DA3420AA	Homo sapiens feces	SAL_JA4592AA_AS	SAL_DA2451AA	Swine
SAL_JA6715AA_AS	SAL_DA3427AA	Homo sapiens feces	SAL_JA4594AA_AS	SAL_DA2586AA	Swine
SAL_JA4794AA_AS	SAL_DA3437AA	Homo sapiens feces	SAL_JA4601AA_AS	SAL_DA2666AA	Swine
SAL_JA0301AA_AS	SAL_DA3444AA	Homo sapiens feces	SAL_JA4605AA_AS	SAL_DA2712AA	Swine
SAL_JA4529AA_AS	SAL_DA3463AA	Homo sapiens feces	SAL_JA4607AA_AS	SAL_DA2942AA	Swine
SAL_JA4540AA_AS	SAL_DA3478AA	Homo sapiens feces	SAL_JA4709AA_AS	SAL_DA3018AA	Swine
SAL_JA4795AA_AS	SAL_DA3499AA	Homo sapiens feces	SAL_JA4710AA_AS	SAL_DA3207AA	Swine
SAL_JA6899AA_AS	SAL_DA3507AA	Homo sapiens feces	SAL_JA4713AA_AS	SAL_DA3851AA	Swine
SAL_JA7000AA_AS	SAL_DA3512AA	Homo sapiens feces	SAL_JA4714AA_AS	SAL_DA3904AA	Swine

The genomes of *Salmonella* Typhimurium used in the phylogenetic analysis. (5)

Genome sequence name	Uberstrain	Sample source	Genome sequence name	Uberstrain	Sample source
SAL_JA7150AA_AS	SAL_DA3550AA	Homo sapiens feces	SAL_JA4716AA_AS	SAL_DA3910AA	Swine
SAL_JA7137AA_AS	SAL_DA3555AA	Homo sapiens feces	SAL_JA4800AA_AS	SAL_DA4198AA	Swine
SAL_JA6851AA_AS	SAL_DA3558AA	Homo sapiens feces	SAL_JA4804AA_AS	SAL_DA4637AA	Swine
SAL_JA6586AA_AS	SAL_DA4750AA	Homo sapiens feces	SAL_JA4817AA_AS	SAL_DA5132AA	Swine
SAL_JA0409AA_AS	SAL_DA4754AA	Homo sapiens feces	SAL_JA4820AA_AS	SAL_DA5233AA	Swine
SAL_JA6730AA_AS	SAL_DA4755AA	Homo sapiens feces	SAL_JA4832AA_AS	SAL_DA5630AA	Swine
SAL_JA6445AA_AS	SAL_DA4822AA	Homo sapiens feces	SAL_JA4851AA_AS	SAL_DA5745AA	Swine
SAL_JA6658AA_AS	SAL_DA4823AA	Homo sapiens feces	SAL_JA4863AA_AS	SAL_DA5981AA	Swine
SAL_JA7016AA_AS	SAL_DA4824AA	Homo sapiens feces	SAL_JA4864AA_AS	SAL_DA6190AA	Swine
SAL_JA6854AA_AS	SAL_DA4826AA	Homo sapiens feces	SAL_JA4879AA_AS	SAL_DA6265AA	Swine
SAL_JA6588AA_AS	SAL_DA4827AA	Homo sapiens feces	SAL_JA4881AA_AS	SAL_DA6418AA	Swine
SAL_JA6852AA_AS	SAL_DA4828AA	Homo sapiens feces	SAL_JA4912AA_AS	SAL_DA6424AA	Swine
SAL_JA6603AA_AS	SAL_DA4829AA	Homo sapiens feces	SAL_JA4913AA_AS	SAL_DA6536AA	Swine
SAL_JA6726AA_AS	SAL_DA4831AA	Homo sapiens feces	SAL_JA4926AA_AS	SAL_DA6648AA	Swine
SAL_JA6572AA_AS	SAL_DA4832AA	Homo sapiens feces	SAL_JA4929AA_AS	SAL_DA6754AA	Swine
SAL_JA6460AA_AS	SAL_DA4833AA	Homo sapiens feces	SAL_JA4937AA_AS	SAL_DA6781AA	Swine
SAL_JA4516AA_AS	SAL_DA4837AA	Homo sapiens feces	SAL_JA4951AA_AS	SAL_DA6785AA	Swine
SAL_JA4985AA_AS	SAL_DA4839AA	Homo sapiens feces	SAL_JA5044AA_AS	SAL_DA7093AA	Swine
SAL_JA6855AA_AS	SAL_DA4841AA	Homo sapiens feces	SAL_JA5047AA_AS	SAL_DA7107AA	Swine
SAL_JA4602AA_AS	SAL_DA4858AA	Homo sapiens feces	SAL_JA5048AA_AS	SAL_DA7254AA	Swine
SAL_JA7109AA_AS	SAL_DA4859AA	Homo sapiens feces	SAL_JA5049AA_AS	SAL_DA7299AA	Swine
SAL_JA7044AA_AS	SAL_DA4862AA	Homo sapiens feces	SAL_JA5051AA_AS	SAL_DA7319AA	Swine
SAL_JA5128AA_AS	SAL_DA4863AA	Homo sapiens feces	SAL_JA5052AA_AS	SAL_DA7337AA	Swine
SAL_JA6690AA_AS	SAL_DA4865AA	Homo sapiens feces	SAL_JA5058AA_AS	SAL_DA7523AA	Swine
SAL_JA6959AA_AS	SAL_DA4867AA	Homo sapiens feces	SAL_JA5064AA_AS	SAL_DA7531AA	Swine
SAL_JA6850AA_AS	SAL_DA4868AA	Homo sapiens feces	SAL_JA5066AA_AS	SAL_DA7533AA	Swine
SAL_JA6518AA_AS	SAL_DA4869AA	Homo sapiens feces	SAL_JA5130AA_AS	SAL_DA7534AA	Swine
SAL_JA6717AA_AS	SAL_DA4911AA	Homo sapiens feces	SAL_JA5131AA_AS	SAL_DA7536AA	Swine
SAL_JA6381AA_AS	SAL_DA4926AA	Homo sapiens feces	SAL_JA5136AA_AS	SAL_DA7540AA	Swine
SAL_BA5403AA_AS	SAL_DA4928AA	Homo sapiens feces	SAL_JA5137AA_AS	SAL_DA7541AA	Swine
SAL_KA0216AA_AS	SAL_EA6698AA	Homo sapiens feces	SAL_JA5138AA_AS	SAL_DA7543AA	Swine
SAL_KA1774AA_AS	SAL_EA8205AA	Homo sapiens feces	SAL_JA5142AA_AS	SAL_DA7547AA	Swine
SAL_KA1775AA_AS	SAL_EA8206AA	Homo sapiens feces	SAL_JA5148AA_AS	SAL_DA7548AA	Swine
SAL_KA1779AA_AS	SAL_EA8210AA	Homo sapiens feces	SAL_JA5180AA_AS	SAL_DA7565AA	Swine
SAL_KA1780AA_AS	SAL_EA8211AA	Homo sapiens feces	SAL_JA5206AA_AS	SAL_DA7568AA	Swine
SAL_KA1782AA_AS	SAL_EA8213AA	Homo sapiens feces	SAL_JA5208AA_AS	SAL_DA7569AA	Swine
SAL_KA1783AA_AS	SAL_EA8214AA	Homo sapiens feces	SAL_JA5209AA_AS	SAL_DA7572AA	Swine
SAL_KA1785AA_AS	SAL_EA8216AA	Homo sapiens feces	SAL_JA5211AA_AS	SAL_DA7573AA	Swine

The genomes of *Salmonella* Typhimurium used in the phylogenetic analysis. (6)

Genome sequence name	Uberstrain	Sample source	Genome sequence name	Uberstrain	Sample source
SAL_KA1789AA_AS	SAL_EA8220AA	Homo sapiens feces	SAL_JA5212AA_AS	SAL_DA7574AA	Swine
SAL_KA1791AA_AS	SAL_EA8222AA	Homo sapiens feces	SAL_JA5214AA_AS	SAL_DA7577AA	Swine
SAL_KA1792AA_AS	SAL_EA8223AA	Homo sapiens feces	SAL_JA5215AA_AS	SAL_DA7579AA	Swine
SAL_KA1797AA_AS	SAL_EA8228AA	Homo sapiens feces	SAL_JA5216AA_AS	SAL_DA7590AA	Swine
SAL_KA1798AA_AS	SAL_EA8229AA	Homo sapiens feces	SAL_JA5217AA_AS	SAL_DA7591AA	Swine
SAL_KA1800AA_AS	SAL_EA8231AA	Homo sapiens feces	SAL_JA5218AA_AS	SAL_DA7829AA	Swine
SAL_KA1802AA_AS	SAL_EA8233AA	Homo sapiens feces	SAL_JA5219AA_AS	SAL_DA7849AA	Swine
SAL_KA1803AA_AS	SAL_EA8234AA	Homo sapiens feces	SAL_JA5224AA_AS	SAL_DA7977AA	Swine
SAL_KA1806AA_AS	SAL_EA8237AA	Homo sapiens feces	SAL_JA5225AA_AS	SAL_DA8093AA	Swine
SAL_HC8683AA_AS	SAL_EA8239AA	Homo sapiens feces	SAL_JA5226AA_AS	SAL_DA8137AA	Swine
SAL_KA1810AA_AS	SAL_EA8241AA	Homo sapiens feces	SAL_JA5228AA_AS	SAL_DA8139AA	Swine
SAL_KA1811AA_AS	SAL_EA8242AA	Homo sapiens feces	SAL_JA5229AA_AS	SAL_DA8424AA	Swine
SAL_KA1812AA_AS	SAL_EA8243AA	Homo sapiens feces	SAL_JA5230AA_AS	SAL_DA8425AA	Swine
SAL_KA1815AA_AS	SAL_EA8245AA	Homo sapiens feces	SAL_JA5233AA_AS	SAL_DA8427AA	Swine
SAL_QA3642AA_AS	SAL_IA3871AA	Homo sapiens feces	SAL_JA5234AA_AS	SAL_DA8442AA	Swine
SAL_UA1845AA_AS	SAL_KA5056AA	Homo sapiens feces	SAL_JA5238AA_AS	SAL_DA8791AA	Swine
SAL_XA9843AA_AS	SAL_NA2727AA	Homo sapiens feces	SAL_JA5239AA_AS	SAL_DA9407AA	Swine
SAL_XA9840AA_AS	SAL_NA2730AA	Homo sapiens feces	SAL_JA5240AA_AS	SAL_DA9510AA	Swine
SAL_XA9766AA_AS	SAL_NA2804AA	Homo sapiens feces	SAL_JA5242AA_AS	SAL_DA9514AA	Swine
SAL_BB9714AA_AS	SAL_QA8080AA	Homo sapiens feces	SAL_JA5246AA_AS	SAL_DA9524AA	Swine
SAL_BB9686AA_AS	SAL_QA8108AA	Homo sapiens feces	SAL_JA5248AA_AS	SAL_DA9527AA	Swine
SAL_CB0349AA_AS	SAL_QA8580AA	Homo sapiens feces	SAL_JA5249AA_AS	SAL_DA9561AA	Swine
SAL_CB0347AA_AS	SAL_QA8582AA	Homo sapiens feces	SAL_JA5261AA_AS	SAL_DA9685AA	Swine
SAL_CB0343AA_AS	SAL_QA8586AA	Homo sapiens feces	SAL_JA5262AA_AS	SAL_DA9692AA	Swine
SAL_CB0340AA_AS	SAL_QA8589AA	Homo sapiens feces	SAL_JA5263AA_AS	SAL_DA9702AA	Swine
SAL_CB0332AA_AS	SAL_QA8597AA	Homo sapiens feces	SAL_JA5282AA_AS	SAL_DA9713AA	Swine
SAL_CB0320AA_AS	SAL_QA8609AA	Homo sapiens feces	SAL_JA5283AA_AS	SAL_DA9738AA	Swine
SAL_CB0787AA_AS	SAL_QA9192AA	Homo sapiens feces	SAL_JA5284AA_AS	SAL_DA9752AA	Swine
SAL_CB0769AA_AS	SAL_QA9210AA	Homo sapiens feces	SAL_JA5285AA_AS	SAL_DA9759AA	Swine
SAL_CB0758AA_AS	SAL_QA9221AA	Homo sapiens feces	SAL_JA5290AA_AS	SAL_DA9767AA	Swine
SAL_GB9934AA_AS	SAL_UA1606AA	Homo sapiens feces	SAL_JA5298AA_AS	SAL_DA9769AA	Swine
SAL_GB9995AA_AS	SAL_UA1612AA	Homo sapiens feces	SAL_JA5299AA_AS	SAL_DA9800AA	Swine
SAL_GB9996AA_AS	SAL_UA1613AA	Homo sapiens feces	SAL_JA5300AA_AS	SAL_DA9890AA	Swine
SAL_GB9998AA_AS	SAL_UA1615AA	Homo sapiens feces	SAL_JA5301AA_AS	SAL_DA9894AA	Swine
SAL_IC0939AA_AS	SAL_UA5721AA	Homo sapiens feces	SAL_JA5401AA_AS	SAL_DA9895AA	Swine
SAL_IC0942AA_AS	SAL_UA5725AA	Homo sapiens feces	SAL_JA5402AA_AS	SAL_DA9932AA	Swine
SAL_IC0943AA_AS	SAL_UA5726AA	Homo sapiens feces	SAL_JA5404AA_AS	SAL_EA0226AA	Swine
SAL_IC0945AA_AS	SAL_UA5728AA	Homo sapiens feces	SAL_JA5406AA_AS	SAL_EA0233AA	Swine

The genomes of *Salmonella* Typhimurium used in the phylogenetic analysis. (7)

Genome sequence name	Uberstrain	Sample source	Genome sequence name	Uberstrain	Sample source
SAL_HC4805AA_AS	SAL_UA5729AA	Homo sapiens feces	SAL_JA5410AA_AS	SAL_EA0238AA	Swine
SAL_IC0946AA_AS	SAL_UA5730AA	Homo sapiens feces	SAL_JA5467AA_AS	SAL_EA0254AA	Swine
SAL_IC0951AA_AS	SAL_UA5735AA	Homo sapiens feces	SAL_JA5507AA_AS	SAL_EA0276AA	Swine
SAL_IC0952AA_AS	SAL_UA5736AA	Homo sapiens feces	SAL_JA5517AA_AS	SAL_EA0277AA	Swine
SAL_IB1695AA_AS	SAL_UA7506AA	Homo sapiens feces	SAL_JA5521AA_AS	SAL_EA0293AA	Swine
SAL_IB1693AA_AS	SAL_UA7508AA	Homo sapiens feces	SAL_JA5522AA_AS	SAL_EA0316AA	Swine
SAL_IB1692AA_AS	SAL_UA7509AA	Homo sapiens feces	SAL_JA5527AA_AS	SAL_EA0327AA	Swine
SAL_IB1691AA_AS	SAL_UA7510AA	Homo sapiens feces	SAL_JA5549AA_AS	SAL_EA0372AA	Swine
SAL_IB1690AA_AS	SAL_UA7511AA	Homo sapiens feces			

Table S6

Genes upregulated in the SDST group compared to the DDST group in the transcriptome analysis

Gene_id	Gene	SDST readcount	DDST readcount	log_2 Fold Change	pval	padj
STM1143	*csgB*	1343.457691	6.294094663	7.7377	1.25E-44	1.60E-42
STM2402	*yfdZ*	1519.138644	9.901121779	7.2614	4.54E-66	1.10E-63
STM1144	*csgA*	1270.095392	16.21507403	6.2915	3.06E-34	2.86E-32
STM0558	*yfdH*	286.8689795	16.19521644	4.1468	4.34E-35	4.22E-33
STM1595	*srfC*	111.0368099	7.660795625	3.8574	3.13E-18	1.83E-16
STM0559	*rfbI*	208.2820845	21.12922934	3.3012	1.11E-22	7.93E-21
STM1141	*csgE*	117.3703458	12.12820954	3.2746	7.26E-16	3.80E-14
STM4401	*ytfG*	74.393443	8.574135999	3.1171	5.11E-11	2.07E-09
STM1140	*csgF*	92.80068345	10.78798537	3.1047	9.88E-13	4.49E-11
STM1142	*csgD*	910.4685453	117.066211	2.9593	1.01E-27	8.22E-26
STM2548	*asrA*	255.6302439	40.0644669	2.6737	7.52E-08	2.19E-06
STM1428	*ydhC*	4614.442112	794.2307806	2.5385	8.96E-13	4.11E-11
STM0156	-//-	234.3251289	42.75815365	2.4542	2.97E-09	1.01E-07
STM2549	*asrB*	93.36737559	17.54867901	2.4116	5.59E-05	0.0010574
STM1555	-//-	106.9389071	20.76853751	2.3643	4.41E-10	1.66E-08
STM1655	*dbpA*	1124.226278	268.9220417	2.0637	1.49E-10	5.79E-09
STM0871	*ybjM*	983.8530585	241.2339498	2.028	3.84E-19	2.39E-17
STM1949	*yecF*	254.0835272	69.75459385	1.8649	1.84E-06	4.52E-05
STM0183	*folK*	46.87924666	13.0614075	1.8436	0.00019882	0.0032873
STM1594	*srfB*	67.05320752	19.80886276	1.7592	3.95E-05	0.00076755
STM0759	*ybgS*	25937.22282	8104.478512	1.6782	1.31E-16	7.16E-15
STM1946	*uvrC*	2188.417428	689.5675302	1.6661	3.14E-15	1.56E-13
STM3408	*sun*	380.3096897	140.8096967	1.4334	8.54E-09	2.79E-07
STM0416	*ribD*	439.0004381	169.2588874	1.375	2.35E-05	0.0004915
STM3579	*yhhQ*	955.9143787	375.521244	1.348	8.99E-10	3.31E-08
STM4295	*adiY*	1524.530068	599.176674	1.3473	0.0013257	0.016566
STM0385	*yaiC*	645.6024306	254.3615493	1.3438	4.64E-09	1.53E-07
STM1817	*rnd*	425.6222352	167.8326137	1.3426	2.92E-08	8.82E-07
STM2799	*stpA*	682.3791728	274.4053391	1.3143	6.71E-09	2.20E-07
STM2550	*asrC*	108.4856238	44.51202323	1.2852	0.00013685	0.0023673
STM1893	*znuB*	265.4438705	111.9865757	1.2451	2.32E-06	5.58E-05
STM0889	*artQ*	465.1590162	197.168668	1.2383	1.90E-07	5.34E-06
STM0383	*yaiB*	1588.814316	674.0506182	1.237	0.0049322	0.046827
STM0890	*artI*	1146.993711	491.6013034	1.2223	1.40E-08	4.39E-07
STM3840	*rnpA*	181.870137	80.20174497	1.1812	3.89E-05	0.00075941
STM1840	*yobG*	87.50052222	38.72424273	1.1761	0.0010129	0.013344
STM0420	*pgpA*	100.9253846	44.9918606	1.1656	0.00064291	0.0093011
STM0206	*btuF*	96.45857876	43.19827584	1.1589	0.00081655	0.011276
STM3842	*yidC*	3491.747201	1567.282851	1.1557	1.46E-08	4.54E-07
STM3457	*kefB*	606.4879028	272.3040161	1.1553	3.96E-07	1.09E-05
STM0855	-//-	126.2795126	56.86528547	1.151	0.0017324	0.020528
STM2546	*suhB*	6217.23272	2800.545182	1.1506	2.57E-06	6.06E-05
STM0260	*dniR*	10657.71635	4824.966877	1.1433	1.12E-08	3.61E-07
STM1225	*potB*	148.4913175	67.96762828	1.1275	0.0001878	0.0031479
STM2136	*yegQ*	1613.650555	738.9373912	1.1268	8.29E-08	2.40E-06
STM4205	-//-	99.64979156	46.37841916	1.1034	0.0011596	0.014833
STM1776	*prfA*	995.9023182	468.4170854	1.0882	4.88E-07	1.33E-05
STM0384	*psiF*	3615.848629	1706.490593	1.0833	0.0011416	0.01468
STM1706	*yciH*	158.4316288	75.15520573	1.0759	0.00026693	0.0042823
STM0873	*ybjC*	81.28921063	39.10479215	1.0557	0.0039379	0.03955
STM3742	*spoT*	1906.421983	921.456262	1.0489	4.30E-07	1.18E-05
STM3874	*gidA*	1047.163885	511.1255407	1.0347	1.69E-06	4.19E-05
STM0975	*ycaO*	1550.817474	757.5847481	1.0335	8.50E-07	2.25E-05
STM1215	*ycfS*	1429.573602	700.3421684	1.0295	0.00027577	0.0043807
STM4005	*glnG*	283.6044321	140.9420806	1.0088	8.13E-05	0.001486
STM0243	*yaeB*	188.914804	94.09044329	1.0056	0.00033259	0.0051819
STM3114	*speC*	579.533708	288.912878	1.0043	1.06E-05	0.00023638
STM3386	*yhdJ*	197.3839634	98.55123801	1.0021	0.00028879	0.0045727
STM0626	*dpiA*	110.8168062	55.35958137	1.0013	0.0020984	0.023612

Reference

1. Donnenberg, M.S. and Kaper, J.B. (1991) Construction of an eae deletion mutant of enteropathogenic *Escherichia coli* by using a positive-selection suicide vector. *Infect Immun*, **59**, 4310-4317.
2. Metcalf, W.W., Jiang, W., Daniels, L.L., Kim, S.K., Haldimann, A. and Wanner, B.L. (1996) Conditionally replicative and conjugative plasmids carrying lacZ alpha for cloning, mutagenesis, and allele replacement in bacteria. *Plasmid*, **35**, 1-13.
3. Wu, R., Zhao, M., Li, J., Gao, H., Kan, B. and Liang, W. (2015) Direct regulation of the natural competence regulator gene *tfoX* by cyclic AMP (cAMP) and cAMP receptor protein (CRP) in Vibrios. *Sci Rep*, **5**, 14921.
4. Khan, S.R., Gaines, J., Roop, R.M., 2nd and Farrand, S.K. (2008) Broad-host-range expression vectors with tightly regulated promoters and their use to examine the influence of TraR and TraM expression on Ti plasmid quorum sensing. *Appl Environ Microbiol*, **74**, 5053-5062.
